# Metasurface-controlled high-speed tunable external cavity lasers

**DOI:** 10.1126/sciadv.aee1791

**Published:** 2026-08-07

**Authors:** Zahra Basiri, Alessandro Tomasino, Gabriel Jülg, Andrea Lanfranchi, Ileana-Cristina Benea-Chelmus

**Affiliations:** ^1^ Hybrid Photonics Laboratory, École Polytechnique Fédérale de Lausanne (EPFL), CH-1015, Switzerland.; ^2^ Center for Quantum Science and Engineering (EPFL), CH-1015, Switzerland.; ^3^ Department of Chemistry and Industrial Chemistry, University of Genoa, 16146 Genova, Italy.

## Abstract

Tunable lasers are essential for optical communication, spectroscopy, and precision sensing, where fast and stable wavelength control is required. Conventional tuning approaches rely on either current or temperature tuning, mechanical actuation, or bulky external modulators, limiting speed, stability, or integration. Here, we introduce an external cavity semiconductor laser incorporating a resonant electro-optic metasurface as the external mirror, enabling ultrafast frequency and amplitude control within a compact, motion-free platform. The metasurface provides narrowband feedback that ensures single-mode operation and voltage-controlled tuning across a wide current range. We demonstrate mode-hop-free linear frequency tuning with rates up to 180 terahertz per second and frequency excursions reaching 110 megahertz under a 10-volt peak radio-frequency drive. By biasing the laser near threshold, we further achieve intensity modulation efficiencies exceeding 100% at 4 volts. These results establish electro-optic metasurfaces as a powerful route toward compact, high-speed, mode-hop-free tunable lasers for free-space communication, ranging, and high-resolution spectroscopy.

## Teaser

Fast and compact wavelength tuning of an external cavity laser is achieved using an electro-optically active metasurface.

## INTRODUCTION

Compact and efficient control of laser emission is an essential feature in diverse scientific and technological contexts, including laser ranging (*1*, *2*), high-resolution spectroscopy (*3*, *4*), optical communications (*5*–*7*), and three-dimensional (3D) printing (*8*). Since the mid-1970s (*9*), tremendous efforts have gone into the development of external cavity diode laser (ECL) designs with a broad range of configurations optimized for wavelength tunability and spectral purity (*10*–*14*). Early proposals relied predominantly on mechanical tuning, which limited speed and stability. To enable faster, voltage-controlled actuation, electro-optic (EO) crystals such as lithium niobate (*15*) and lithium tantalate (*16*) were later introduced as tuning elements (*17*). However, modulators made from these materials were bulky and required a grating in addition to the modulator to simultaneously achieve single-mode operation and high-speed control over the laser wavelength (*18*). These multicomponent systems were prone to multiple reflections and misalignment over time, ultimately affecting the long-term stability of the entire system.

The lack of compact modulators put a roadblock to reducing the system footprint while maintaining fast and single-mode operation. Recent advances in integrated photonic platforms have begun to propose solutions using on-chip EO and piezoelectric modulators that enable ultrafast tunable lasers (*12*–*14*, *19*). However, similar capabilities have not been achieved for free-space laser systems, where the intracavity light propagates in free space rather than in a waveguide (*20*). In contrast to integrated photonic lasers, these systems would be immediately applicable to free-space applications such as imaging (*21*–*23*), ranging (*1*, *24*–*26*), diffractive optics, and light projection (*27*–*29*). They are also attractive for applications where propagation in fibers or along waveguides can lead to uncontrolled dispersion, nonlinearities, and temperature fluctuations (*30*–*32*).

Optical metasurfaces, i.e., planar arrays of subwavelength elements, condense the functionalities of multiple bulky and complex optical components into a single, engineered 2D surface (*20*, *22*, *33*–*43*). Their compactness makes them particularly important in applications requiring a small footprint, along with improved long-term robustness and stability. In most applications, metasurfaces are used downstream of the laser, where they manipulate the space-time characteristics of the light with minimal component overhead, simplifying an optical setup (*22*). In this case, they do not affect the lasing process.

Metasurfaces can be implemented directly as part of a laser cavity, where their multifunctionality enables custom-tailored and dynamic feedback to the gain medium. This integration allows control over the spatial, spectral, and polarization properties of laser emission (*44*). Two main strategies have emerged for the concrete cavity design. One approach relies on patterning subwavelength structures into the gain medium, effectively transforming it into a metasurface. This has been used to statically shape the beam profile of perovskite lasers (*45*), implement low-threshold semiconductor lasers (*46*, *47*), or control the polarization state of terahertz quantum-cascade lasers (*48*–*51*). A second approach uses metasurfaces as engineered mirrors of the laser cavity, either patterned directly on the laser’s facet or in an external cavity configuration. While static metasurfaces have been patterned directly onto the laser facet to achieve spin-dependent beam shaping (*52*) or mode-selective metasurface vertical cavity surface emitting lasers (*53*), or designing spatially tunable photonic-crystal surface-emitting lasers (*54*–*56*), incorporating a tunable mechanism into these designs remains a tremendous task due to space limitations and fabrication constraints. To address this limitation, external cavities have been proposed. In this case, the reflection is suppressed at one of the laser’s facets with an antireflection coating, and tailoring the external mirror provides flexibility in controlling laser emission. Following this approach, a multifunctional metasurface external cavity laser (*57*) has been proposed, but the lack of electrical tunability necessitated mechanical adjustments for wavelength control.

Meanwhile, several approaches have been explored to make metasurfaces electrically tunable. Among active tuning methods, EO metasurfaces offer fast, voltage-driven control in a compact form (*1*, *26*, *58*–*61*). They often rely on resonant metasurface designs, which exploit narrow resonances as a means to provide reflection or transmission only within a desired frequency band. This makes them uniquely suited for achieving single-mode laser emission. Furthermore, the radiative nature of these resonances can often be controlled through geometry, enabling reflection characteristics with custom-tailored bandwidth. Further integrating Pockels materials within such metasurfaces enables electrical control over these characteristics (*59*, *62*–*66*) at speeds up to gigahertz frequencies (*67*), opening up the intriguing question whether these tunable metasurfaces can facilitate ultrafast tunable lasers.

Here, we demonstrate the integration of an ultrathin EO modulator with a laser medium to simultaneously engineer the optical spectrum and enable high-speed modulation of laser light in an external cavity configuration. By exploiting guided mode resonances, we provide frequency-dependent feedback to a semiconductor laser diode operating in the telecom C-band and achieve single-mode operation. The guided mode resonances form within a single layer of EO material onto which we pattern an array of metallic electrodes, similar to our previous work (*66*). We show that tweaking the array periodicity enables lasing at engineered frequencies across the gain bandwidth, while matching the gain with the resonance frequency of the guided modes influences laser threshold. Enabled by the large EO coefficient of the organic layer, we provide voltage-tunable feedback to the gain medium, enabling control over the laser frequency and output power at megahertz frequencies. Outperforming alternative approaches in terms of modulation speed and compactness, this platform holds strong potential for high-speed optical communication applications.

## RESULTS

### Tunable external cavity concept

**Fig. 1.**
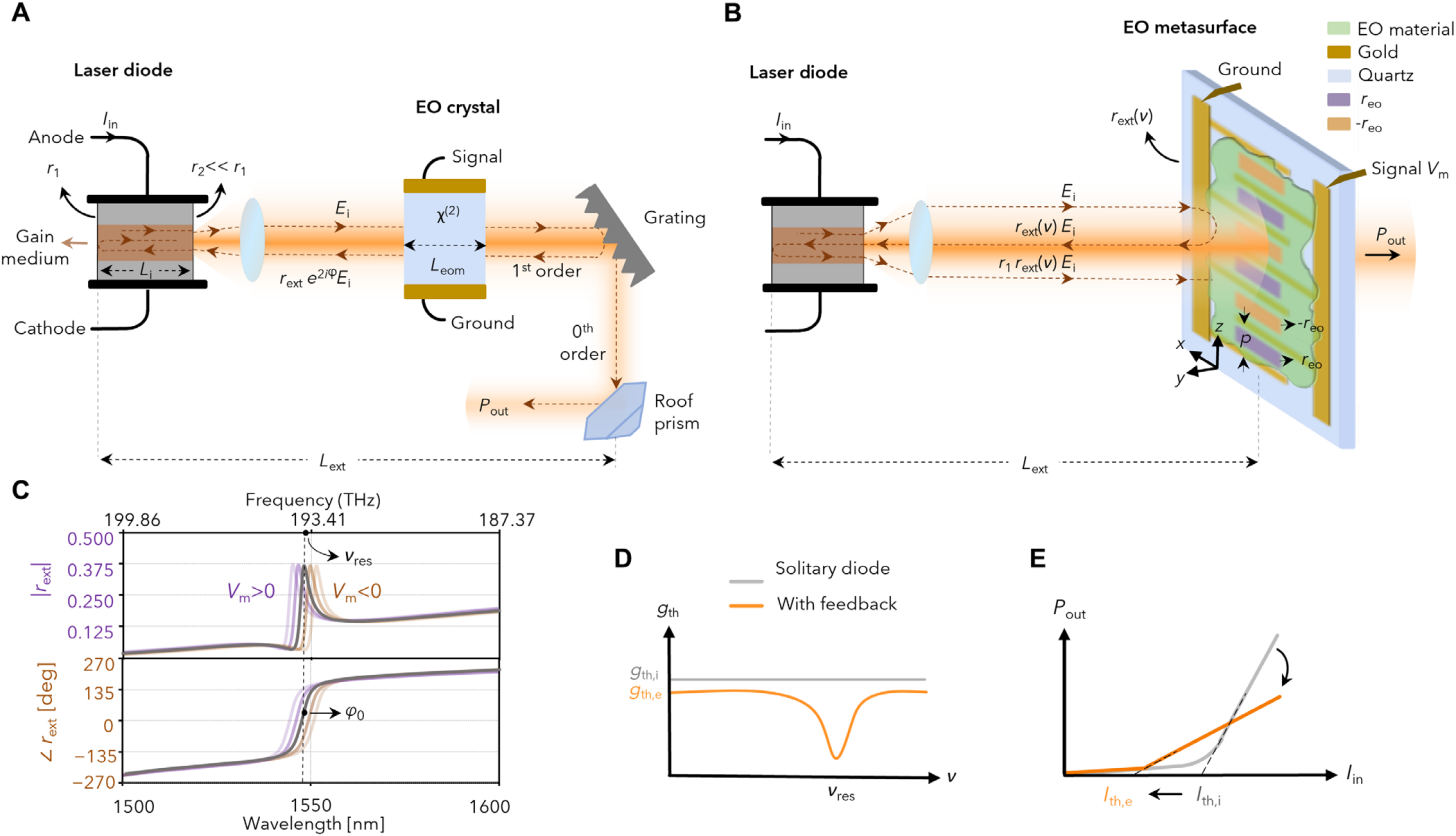
Working principle of the metasurface-assisted tunable external cavity. (**A**) Conventional Littrow configuration with EO modulation: The injection current $I_{\text{in}}$ supplies carriers to the gain medium of the laser diode. The back facet has reflection $r_{1}$, while the front facet is antireflection coated with $r_{2}\ll r_{1}$. For EO wavelength control, an EO crystal is placed in the external cavity and modulates the optical path length via the Pockels effect. An applied electric field induces a refractive index change in the crystal, which results in a modulation of the optical field $E_{i}$ inside the cavity as $e^{2i\varphi }E_{i}$, where φ is the EO phase shift induced by the modulator. Afterward, the beam is diffracted by the grating. The first-order diffraction provides coherent feedback to the cavity via $r_{\text{ext}}$, while the zeroth-order serves as the laser output. (**B**) Metasurface-controlled external cavity laser: The metasurface condenses the reflection grating and the EO control. The reflection from the metasurface is frequency-selective, exhibiting a resonance around $\nu_{\text{res}}$, with a maximum value $r_{\text{max}}\gg r_{1}$. An electric field applied to the metasurface modifies its refractive index and hence resonance, yielding control over the lasing frequency. (**C**) The simulation illustrates the resonant behavior of the EO metasurface (top graph shows the reflection amplitude, and bottom graph the corresponding phase response). Applying a voltage to the metasurface modifies both the amplitude and phase of the light circulating in the cavity, as illustrated by semitransparent curves. (**D**) For the solitary diode, the lasing threshold is determined solely by its length and gain bandwidth. When the metasurface provides frequency-selective feedback, the effective gain threshold decreases near the resonance. (**E**) A reduced gain threshold results in a lower required injection current for lasing, from $I_{\text{th},i}$ to $I_{\text{th}}$. This simultaneously decreases the output power $P_{\text{out}}$ through decreased slope efficiency.

One of the simplest and most widely used external cavity setups is the Littrow configuration. Here, light emitted from a laser diode is collimated and directed onto a diffraction grating (*18*). The first-order diffracted beam is reflected back into the diode to provide optical feedback, while the zeroth-order beam serves as the output (Fig. 1A). In this configuration, adjusting the laser’s wavelength is possible only by rotating the grating, which is slow and requires careful alignment. To overcome these speed limits, earlier ECL designs incorporated bulk EO crystals directly into the external cavity (*15*–*17*). In this configuration, applying a voltage across the EO crystal alters its refractive index via the Pockels effect, thereby modifying the optical path length of the cavity and consequently the lasing frequency. The corresponding frequency tuning can be expressed as $\Delta \nu =-\nu \frac{L_{\text{eom}}}{L_{\text{ext}}}\frac{\Delta n_{\text{eo}}}{n}$, where $L_{\text{eom}}$ is the length of the EO modulator, $\frac{\Delta n_{\text{eo}}}{n}$ the relative refractive index change, and $L_{\text{ext}}$ is the total cavity length (*68*). This relation shows that the achievable frequency shift decreases with increasing external cavity length. Consequently, minimizing the size of the external cavity is desirable to maximize the total frequency shift. However, the large size of commercial EO modulators was a roadblock toward achieving short cavity lengths (*17*), resulting in applied voltages reaching hundreds of volts to maximize frequency excursion.

Guided by our goal to achieve large frequency excursion at lower voltages, we reduce the footprint of the external cavity by condensing the bulk EO crystal and the diffraction grating into a single, compact, frequency-selective EO metasurface with custom-tailored reflectivity $r_{\text{ext}}\left(\nu \right)$ (Fig. 1B). In this case, light generated and emitted from the laser diode $E_{i}$ by applying the injection current $I_{\text{in}}$ impinges directly onto the EO device after being collimated by a lens and is partially reflected back into the cavity $r_{\text{ext}}\left(\nu \right)E_{i}$. The back-propagating field then couples into the active region of the laser diode, where it is reflected by the internal mirror $r_{1}r_{\text{ext}}\left(\nu \right)E_{i}$, establishing a full round-trip (indicated by the brown dashed line). The reflection from the emitting facet is neglected since its reflectivity $r_{2}$ is much weaker than the one provided by the EO metasurface.

The EO metasurface consists of a periodic array of gold stripes with pitch size *p* patterned on a quartz substrate onto which we spin-coat a layer of the nonlinear molecules JRD1 (*65*) mixed with polymethylmethacrylate (PMMA), similar to our recent demonstration (*66*) (details about the geometry are given in fig. S2A). The molecules exhibit a $\chi^{\left(2\right)}$ nonlinearity, replacing thereby the EO crystal used in the conventional configuration of Fig. 1A (*15*–*17*). The combination of the slab formed by the nonlinear material and the interdigitated gold stripes supports guided-mode resonances (*69*), which occur when the diffraction angles of the gold stripes coincide with the total internal reflection angle of the guided modes. This enables a frequency-selective reflection $r_{\text{ext}}\left(\nu \right)$, with a maximum value $r_{\text{max}}$ occurring at a specific resonance frequency $\nu_{\text{res}}$ and corresponding to the resonance wavelength $\lambda_{\text{res}}=c/\nu_{\text{res}}$, replacing the grating of Fig. 1A. By engineering the thickness of the slab and the periodicity of the grating, these guided-mode resonances can be so sparse that a single resonance occurs across the entire gain bandwidth of the laser diode, spanning more than 100 nm, with no other resonances nearby (Fig. 1C). This ensures feedback on a well-defined resonance and prevents mode hopping. We note that this extremely high free-spectral range (exceeding 10 THz) is a unique feature of subwavelength resonances, in stark contrast to approaches using high modal number resonators, such as ring resonators, which feature much lower free-spectral ranges (tens to hundreds of gigahertz) and consequently need Vernier filtering to prevent mode hopping. Details about the design of the EO modulator can be found in section S3.

We use the EO effect provided by the JRD1 molecules as a tuning knob for achieving voltage-controlled laser emission. However, upon spin-coating, the nonlinear molecules are not electro-optically active. They are activated through electric field poling, in which the highly polarizable molecules are oriented along a poling field, producing periodically poled domains with EO coefficients alternating between $-r_{\text{eo}}$ and $+r_{\text{eo}}$ in adjacent periods. This requires applying a strong dc voltage to the molecules via the interdigitated arrays and heating the sample above glass transition temperature (the full protocol is provided in the section S1.1). After activation, applying a radio-frequency field of voltage $V_{m}$ to the interdigitated electrodes tunes the refractive index $n_{\text{nlm}}$ of the entire external mirror by $\Delta n_{\text{eo}}\left(t\right)=\frac{1}{2}n_{\text{nlm}}^{3}r_{\text{eo}}\frac{V_{m}\left(t\right)}{p}$ (*66*) through the Pockels effect. This, in turn, allows controlling the phase and magnitude of the reflection for a given frequency ν (Fig. 1C, section S4, and fig. S9). Simulation results in Fig. 1C were obtained using a frequency domain solver implemented in CST Studio Suite, unit cell module with periodic boundary conditions. Further details are provided in the section S3.

In line with laser theory, the magnitude and the frequency dependence (Fig. 1C) of the reflection of the EO device, $r_{\text{ext}}\left(\text{ν}\right)$, influence several key lasing parameters. First, only those modes that experience sufficiently high reflection start lasing, showcasing how a resonant design can achieve narrowband lasing. This is because from the viewpoint of the laser diode, a narrowband reflection lowers the effective gain threshold only in a custom tailored band, in contrast to the solitary antireflection-coated laser diode, which has a high gain threshold across the entire gain bandwidth (orange and gray curves in Fig. 1D). Second, the magnitude of the reflection peak determines how much the threshold current, $I_{\text{th}}$, decreases compared to the solitary laser diode since $I_{\text{th}}\left(\nu \right)\propto \frac{-\text{ln}\left[r_{1}r_{\text{ext}}\left(\nu \right)\right]}{2L_{i}}$, where $L_{i}$ is the length of the internal cavity and $r_{1}$ is the reflectivity of the laser facet. The threshold current describes the minimal current at which lasing occurs. Third, the magnitude of the reflection peak also determines the amount by which the slope efficiency $\eta_{\text{slope}}$ decreases (Fig. 1E), since $\eta_{\text{slope},I>>I_{\text{th}}}\propto \left[1-r_{1}r_{\text{ext}}\left(\nu \right)\right]$. The slope efficiency describes the increase of output power versus current (*70*). Consequently, while the feedback improves the lasing conditions, it also reduces the output power per unit of injection current above the threshold.

### Spectral shaping

**Fig. 2.**
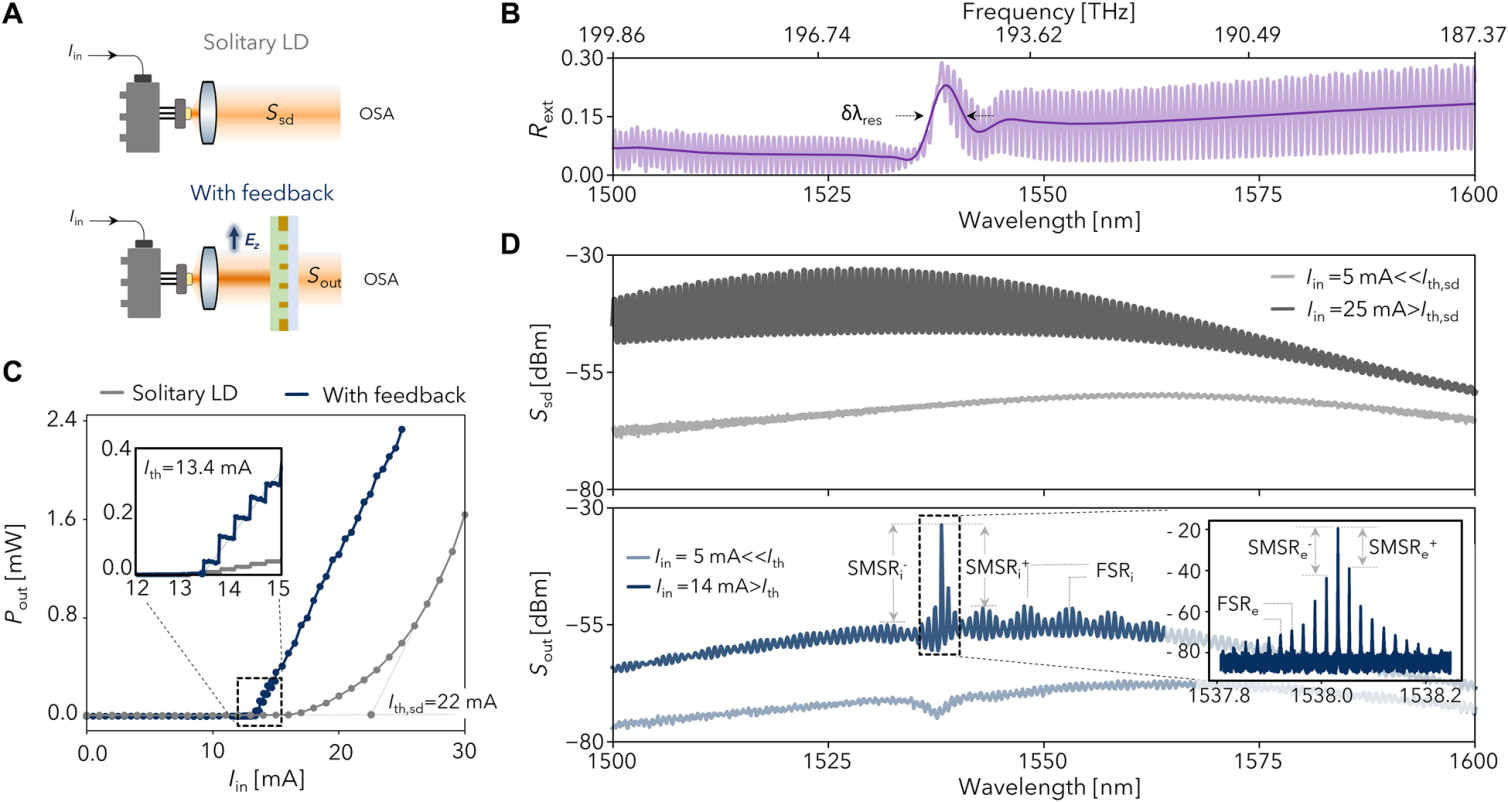
Metasurface-controlled laser diode feedback. (**A**) To characterize the two lasing configurations, the optical spectrum is sent directly to an optical spectrum analyzer (OSA), and the output power is monitored with a power meter. The EO metasurface is then placed in the external cavity and aligned such that the electric field inside the cavity is oriented perpendicular to the gold stripes of the metasurface. (**B**) The reflectance spectrum of the metasurface is plotted. The maximum reflectance, $R_{\text{max}}=0.28$, occurs at the resonance wavelength, with a linewidth of ${\delta \lambda }_{\text{res}}=7$ nm. (**C**) The solitary diode shows a lasing threshold of $I_{\text{th},\text{sd}}=22$ mA (gray curve). Once optical feedback is established, the lasing threshold is reduced to $I_{\text{th}}=13.4$ mA. (**D**) Spectral response with and without feedback. Top panel shows the emission spectrum of the solitary laser diode, denoted $S_{\text{sd}}$. Below threshold (light gray), at $I_{\text{in}}=5$ mA, and above threshold, at $I_{\text{in}}=25$ mA, the laser exhibits spontaneous and stimulated emission, respectively. The gain spectrum is ∼80 nm wide, with a noticeable blue shift of ∼32 nm as the current increases. Bottom panel shows the emission spectra in the presence of feedback, denoted $S_{\text{out}}$, shown both below threshold at $I_{\text{in}}=5$ mA (light blue) and above threshold at $I_{\text{in}}=14$ mA (dark blue). Below threshold, the resonance of the metasurface appears as a dip in transmission. Above threshold, lasing occurs at the resonance, and off-resonant modes are suppressed. The internal and external cavity mode spacings are marked as ${\text{FSR}}_{i}$ and ${\text{FSR}}_{e}$, respectively. The differences in intensity between the main lasing peak and the adjacent internal and external sidebands are quantified as ${\text{SMSR}}_{i}$ and ${\text{SMSR}}_{e}$, respectively. The zoomed-in view of the dominant lasing peak reveals the external cavity mode structure.

Guided by these considerations, we now investigate the impact of the design of the EO device on the electrical and optical characteristics of the external cavity laser by comparing it to the solitary laser diode (Fig. 2A). Its anti reflection coated facet has a reflectance of $R_{1}\leq 5\times 10^{-4}$ over the measurement wavelength range. We select a device with a pitch size of p=1.04 μm, which provides a reflectance whose peak aligns well with the gain peak of the laser diode. The peak reflectance is $R_{\text{max}}\approx 0.28$ at $\lambda_{\text{res}}=1538$ nm and the linewidth of the resonance is ${\delta \lambda }_{\text{res}}\approx 8$ nm (Fig. 2B). Fabry-Pérot resonances from the glass substrate are visible in the raw reflectance curve shown in light purple, while the filtered reflectance is shown in darker purple for clarity. Using this device in the external cavity configuration lowers the threshold current notably to $I_{\text{th}}=13.4$ mA, compared to the solitary laser diode with $I_{\text{th},\text{sd}}=22$ mA, indicating successful optical feedback between the laser diode and the metasurface (Fig. 2D). Furthermore, the threshold is markedly more abrupt in the external cavity configuration, as expected. Step-like features appear in the light-current curve in both configurations due to mode hopping: As the injection current varies, the longitudinal modes of the laser shift until a different mode satisfies the resonance condition, producing small yet abrupt changes in output power, in line with existing literature (*70*–*74*) (inset of Fig. 2C). In the external cavity configuration, these hops occur when the internal and external cavity modes detune and then realign. Last, we also inspect the beam shape for the two configurations and find no pronounced features originating from the metasurface (see section S5.5).

The spectral characteristics of laser emission are also markedly different for the two configurations (Fig. 2D). First, the AR-coated solitary laser diode features multimode emission (Fig. 2D, top), while the external cavity laser features single-mode operation (Fig. 2D, bottom). Second, while the most intense modes follow the gain peak in the case of the solitary laser diode, this is not the case for the external cavity configuration where the lasing frequency aligns with the maximum of the reflection provided by the EO device, as visible when comparing the reflectance peak in Fig. 2B and the lasing mode in the bottom panel of Fig. 2D. We note that below the threshold, the resonance of the EO device appears as a dip in the luminescence spectra, since less power is out-coupled from the cavity in this band (gray curve in Fig. 2D). Third, this frequency-locking is observed over a wide range of currents (see section S5.2) and mitigates the typical blue-shift of the lasing peak, which is present in laser diodes featuring broadband reflection characteristics (see blue-shift between peak of luminescence spectra below threshold and peak of lasing spectra above threshold for the solitary laser diode in Fig. 2D and fig. S14B). This well-known behavior in III-V semiconductor lasers is driven by the combined effects of band-filling and quasi-Fermi level separation dynamics (*75*, *76*). Together, this showcases that our approach combines the advantages of conventional external-cavity diode configurations, such as Littrow and Littman (*18*) in frequency locking, while offering a compact solution.

The linewidth of the resonance determines, together with the free spectral range of the external cavity, the degree of suppression of side modes, known as side-mode suppression ratio (SMSR). For an effective suppression, the linewidth needs to be commensurate with the free spectral range. For a mode to exist in the external cavity, the total phase accumulation $\varphi_{\text{tot}}$ during one round trip in the cavity is an integer multiple of 2π, as

$$\varphi_{\text{tot}}=2k_{0}\left[\left(n_{c}-1\right)L_{i}+L_{\text{ext}}\right]+\varphi_{0}=2\pi m$$ (1)

leading to mode formation at frequencies $\nu_{m}=\frac{\left(m-\frac{\varphi_{0}}{2\pi }\right)c_{0}}{2\left[\left(n_{c}-1\right)L_{i}+L_{\text{ext}}\right]}$, where $n_{c}$ is the refractive index of the cavity, $k_{0}$ is the vacuum wavenumber, $\varphi_{0}$ is the phase at the resonance peak of the metasurface, and *m* is the mode number. We choose an external cavity length of $L_{\text{ext}}=2.5$ cm, corresponding to external cavity modes that are mutually separated by approximately ${\text{FSR}}_{e}=5$ GHz. This cavity length ensures that the laser operates in single mode with a minimal SMSR of external modes of ${\text{SMSR}}_{e}\approx 25$ dB, as revealed in the zoom-in in the bottom panel of Fig. 2D. The internal cavity modes with a free spectral range of ${\text{FSR}}_{i}=90$ GHz are suppressed by up to ${\text{SMSR}}_{i}\approx 40$ dB (inset of Fig. 2D). In section S5.2, we show that these internal and external sideband suppression ratios, ${\text{SMSR}}_{i}$ and ${\text{SMSR}}_{e}$, are consistent across various injection currents, ensuring controlled laser operation across a large dynamic range.

The exact lasing wavelength can be controlled by adjusting the pitch size of the interdigitated electrode array, with smaller pitch sizes leading to a blue shift in the laser emission (see section S3.1). We showcase this tunability by fabricating and measuring two different EO devices with pitch values of p=1.04 μm and p=1.06 μm, featuring $\lambda_{\text{res}}=1539$ nm and $\lambda_{\text{res}}=1559$ nm, respectively (see section S5.1). We chose these values to study the impact of detuning of the EO device’s resonant frequency with the gain maximum on the optical and electrical characteristics of the laser. When comparing these two devices, we find a greater reduction in threshold current for the devices with $\lambda_{\text{res}}=1559$ nm, although its reflectance is lower. This demonstrates that feedback effectiveness depends not only on the reflectance magnitude, but also on the spectral alignment between the external cavity resonance and the laser diode gain profile. If desired, such shifts in the lasing frequency can be achieved within the same platform by exploiting the angular tuning of guided mode resonances (controlled in-plane rotation/tilt), while maintaining cavity alignment through beam steering (*66*).

We model this behavior by solving the rate equations for a wavelength-dependent reflectivity (section S4.1). By studying various detuning conditions between the cavity resonant frequency $\nu_{\text{res}}$ and the central gain frequency $\nu_{0}$, we confirm that the threshold current is lowest and the output power is highest at zero detuning. Overall, our observations demonstrate the importance of co-designing the EO device, the laser cavity, and the laser diode for achieving optimal electrical and optical characteristics of the entire system.

### EO frequency tuning

Building on the static spectral control provided by the metasurface, we now exploit its dynamic tuning capability via the EO effect. Upon applying an RF voltage $V_{m}$ to the metasurface, its refractive index is modified by $\Delta n_{\text{eo}}$, resulting in a shift of its resonance frequency $\Delta \nu_{\text{res}}$ of

$$\frac{\Delta \nu_{\text{res}}}{\nu_{\text{res}}}=-\frac{\Delta \lambda_{\text{res}}}{\lambda_{\text{res}}}=-\frac{\Delta n_{\text{eo}}}{n_{\text{nlm}}}\Gamma_{c}$$ (2)

where $\Gamma_{c}$ is the overlap factor between the optical field and the RF field within the nonlinear material (*66*). In the small signal approximation, this shift in the metasurface resonance influences laser emission through modifying the reflection phase at the external mirror $\varphi_{0}$, and hence the round-trip condition at a given wavelength, rather than the absolute value of the reflection $r_{\text{max}}$, and hence the gain threshold. This is because the operation point of the laser coincides with the point of maximal reflection, where the roundtrip losses are lowest. Here, the rate of change of the phase with voltage $\frac{\partial \varphi_{0}}{\partial V_{m}}=\frac{\partial \varphi }{\partial V_{m}}{|{}_{\nu_{\text{res}}}=\frac{\partial \varphi }{\partial \nu_{\text{res}}}\frac{\partial \nu_{\text{res}}}{\partial V_{m}}|}_{\nu_{\text{res}}}$ is maximal since ${\frac{\partial \varphi }{\partial \nu_{\text{res}}}|}_{\nu_{\text{res}}}$ is maximal while the rate of change of the magnitude $\frac{\partial r_{\text{ext}}}{\partial V_{m}}{|{}_{\nu_{\text{res}}}=\frac{\partial r_{\text{ext}}}{\partial \nu_{\text{res}}}\frac{\partial \nu_{\text{res}}}{\partial V_{m}}|}_{\nu_{\text{res}}}$ is zero since ${\frac{\partial r_{\text{ext}}}{\partial \nu_{\text{res}}}|}_{\nu_{\text{res}}}=0$. This fundamental property of the metasurface-controlled external cavity laser we propose is essential, as it results in a frequency modulation of laser emission and minimizes intensity modulation. Such decoupling of frequency modulation from intensity modulation is highly desirable in most applications, including communications and ranging applications. The exact amount of amplitude modulation is measured in the last section of this manuscript.

The amount of frequency modulation $\Delta \nu_{m,V=V_{m}}$ at voltage $V_{m}$ can be retrieved by re-evaluating the round-trip condition upon a change in the phase of the reflected electric field (from its original value $\varphi_{0}$ to $\varphi_{0}+\Delta \varphi_{\text{eo}}$)

$$\Delta \nu_{m,V=V_{m}}=\frac{c_{0}\Delta \varphi_{\text{eo}}}{4\pi \left[\left(n_{c}-1\right)L_{i}+L_{\text{ext}}\right]}$$ (3)

Using the relationships above, the amount of phase modulation $\Delta \varphi_{\text{eo}}$ depends on the shift of the resonance $\Delta \nu_{\text{res}}$ and on the steepness of the phase modulation around the resonance ${\frac{d\varphi }{d\nu_{\text{res}}}|}_{\nu_{\text{res}}}$, yielding ${\Delta \varphi_{\text{eo}}\approx \frac{d\varphi }{d\nu_{\text{res}}}|}_{\nu_{\text{res}}}\Delta \nu_{\text{res}}$. This underlines the importance of having both high quality-factor resonances (yielding large steepness) and a strong EO response (yielding large shifts of the resonance frequency upon an applied bias) for achieving large frequency excursions. We quantify these aspects using finite element methods (see section S3.4). Together, these considerations result in an EO-induced shift in the lasing frequency that depends linearly on the shift in the resonance frequency, $\Delta \nu_{\text{res}}$, and hence in applied voltage $V_{m}$, and inversely on the length of the external cavity, $L_{\text{ext}}$ (see Materials and Methods)

$${\Delta \nu_{m,V=V_{m}}=\frac{c_{0}}{4\pi \left[\left(n_{c}-1\right)L_{i}+L_{\text{ext}}\right]}\frac{d\varphi }{d\nu_{\text{res}}}|}_{\nu_{\text{res}}}g_{\text{eo}}V_{m}$$ (4)

where $g_{\text{eo}}=\frac{\nu_{\text{res}}\;n_{\text{nlm}}^{3}r_{\text{eo}}\Gamma_{c}}{2d}$ is a proportionality constant that characterizes the EO response of the nonlinear material to the applied $V_{m}\left(t\right)$. A higher quality-factor resonance is beneficial, as its narrower optical linewidth $\delta \nu_{\text{res}}$ supports larger frequency excursion at lower values of the RF modulation voltage $V_{m}$.

**Fig. 3.**
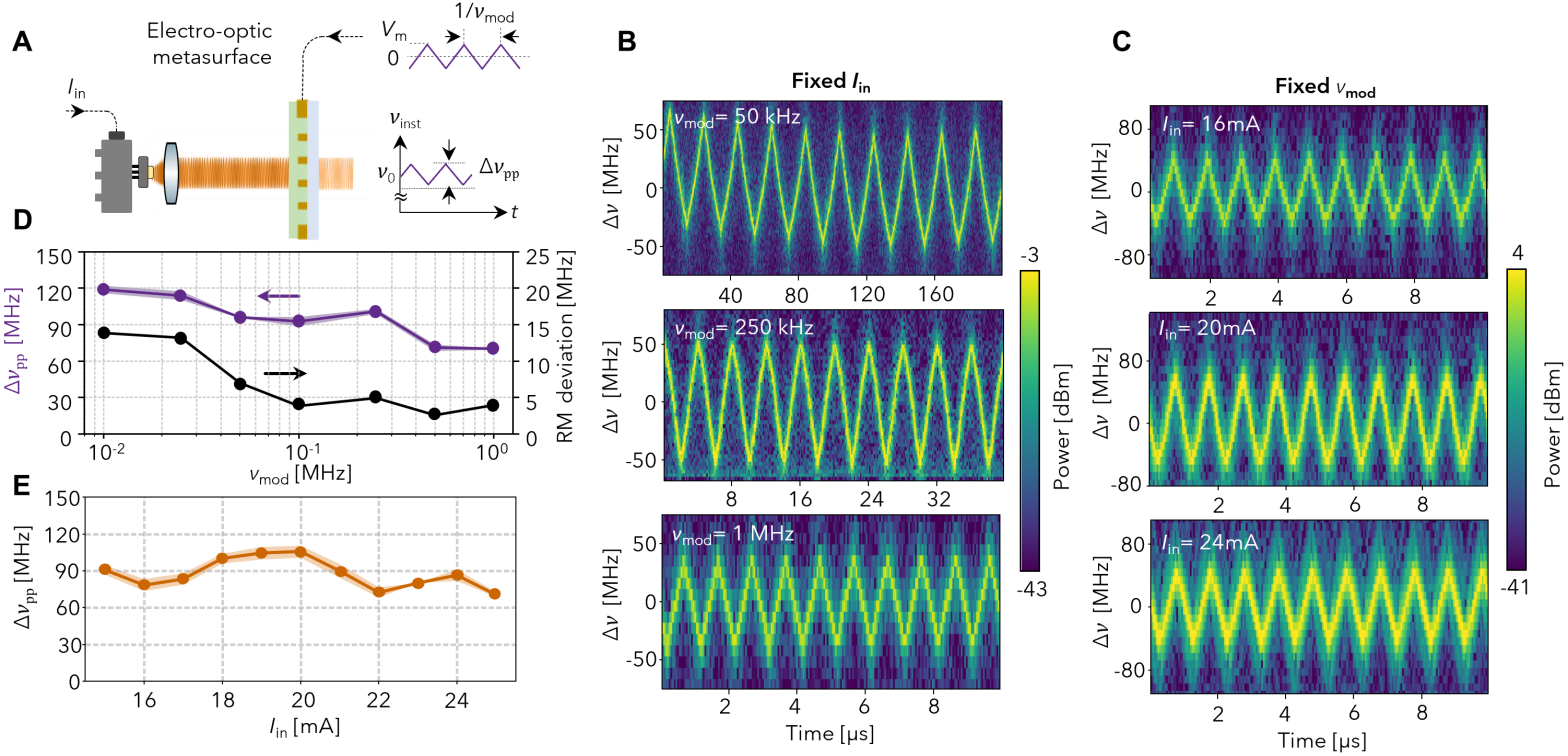
Ultrafast frequency modulation of a laser diode with an EO metasurface. (**A**) An RF source applies a triangular voltage waveform with peak amplitude $V_{m}$ and frequency $\nu_{\text{mod}}$ to the metasurface, dynamically tuning its reflection. This modulation alters the lasing frequency of the diode laser. The output beam is combined with a frequency-stable reference laser in a heterodyne detection setup, enabling extraction of the instantaneous frequency $\nu_{\text{inst}}\left(t\right)$ through RF beatnote analysis. The modulation induces a frequency excursion Δν centered around a carrier frequency $\nu_{0}$. (**B**) Spectrograms of the heterodyne beatnote signal under triangular RF modulation at three different frequencies: 50 kHz, 250 kHz, and 1 MHz. All measurements were performed at a fixed RF amplitude of $V_{m}=10$ V and injection current $I_{\text{in}}=16$ mA. The color scale indicates optical power, calibrated to the output power from the laser’s light-current (LI) curve. (**C**) Heterodyne spectra measured at a fixed modulation frequency $\nu_{\text{mod}}=1$ MHz for injection currents $I_{\text{in}}=16$, 20, and 24 mA. The traces illustrate how the frequency excursion and heterodyne signal amplitude evolve with injection current. As the current increases, the frequency excursion gradually narrows. The amplitude of the beatnote is calibrated using the laser’s LI curve. (**D**) The left vertical axis (purple) shows the peak-to-peak frequency excursion $\Delta \nu_{\text{pp}}$, with the shaded region representing the standard deviation across modulation cycles. The right axis (black) shows the RMS deviation from an ideal triangular waveform. As $\nu_{\text{mod}}$ increases, the frequency excursion decreases while the RMS deviation improves, illustrating the trade-off between modulation speed and dynamic range. (**E**) Frequency excursion as a function of injection current $I_{\text{in}}$ at fixed modulation frequency $\nu_{\text{mod}}=1$ MHz. The shaded area indicates the standard deviation of the measured excursion.

We demonstrate EO resonance tuning by driving the metasurface with a triangular RF voltage of amplitude $V_{m}=10$ *V* and frequency $\nu_{\text{mod}}$ in the configuration shown in Fig. 3A. The laser’s instantaneous frequency response is monitored via heterodyne detection. The time-resolved frequency excursion Δν(t) is extracted from the beating between the modulated laser and a reference laser. The detailed setup is presented in fig. S9. We find that the instantaneous laser frequency follows closely the triangular shape of the modulation voltage, as illustrated in the spectrograms obtained for modulation frequencies of $\nu_{\text{mod}}=50$ kHz, 250 kHz, and 1 MHz (Fig. 3B). This linear dependence on applied voltage is consistent with Eq. 4. For an applied peak voltage amplitude of $V_{m}=10$ V and constant injection current $I_{\text{in}}=16$ mA, frequency excursions of ∼80, 90, and 60 MHz are observed at these three modulation frequencies, respectively. These values remain consistent across various injection currents, including further above threshold: $I_{\text{in}}=16$, 20, and 24 mA, for a modulation frequency of $\nu_{\text{mod}}=1$ MHz, where the frequency excursion remains nearly constant between 70 and 90 MHz (Fig. 3C). The absolute optical powers of the laser emission are retrieved by calibrating the intensity of the heterodyne signal with the *I*-*V* curve measurement shown in Fig. 2C.

When analyzing the peak-peak frequency excursion $\Delta \nu_{\text{pp}}$ as a function of modulation frequency, somewhat larger excursions are observed at lower modulation frequencies (purple curve, Fig. 3D). Furthermore, since an accurate prediction of the instantaneous frequency is highly relevant in practical applications, we investigate the root mean square (RMS) deviation of the instantaneous frequency from the ideal triangular waveform. Details of the RMS calculation method and its interpretation are provided in section S5.3 and visualized in fig. S16. The RMS deviation metric quantifies the fidelity of the frequency chirp to the applied triangular drive signal and should not be confused with the instantaneous linewidth, which is extracted separately from heterodyne beat measurements. We find the instantaneous linewidth of our external cavity laser to be about 1.8 MHz (see section S5.4).

While at higher modulation frequencies, the RMS deviation is below 3 MHz (in line with the laser’s instantaneous linewidth), and it increases at lower modulation frequencies, where the longer scan duration increases the sensitivity of the system to slow environmental fluctuations affecting the external cavity length. Such slow oscillations are well known in external-cavity laser diode systems and originate from acoustic, thermal, and mechanical perturbations. These effects can be mitigated through improved mechanical rigidity, vibration isolation, thermal stabilization, or active cavity-length stabilization. In contrast, we note that the reduced spectral resolution decreases the accuracy of the RMS calculation at higher frequencies. At a constant modulation frequency of $\nu_{\text{mod}}=1$ MHz, the excursion remains between 70 and 120 MHz for various injection currents, as shown in Fig. 3E. Shaded regions indicate the standard deviation. These results are in line with estimated values of the frequency excursion for our particular metasurface design, demonstrating the validity of our approach (see section S3.4). Higher modulation frequencies could, in principle, be explored; however, in the present analysis, they are limited by the time-frequency resolution of the heterodyne short-time Fourier transform method used to extract the instantaneous frequency, rather than by the intrinsic EO bandwidth of the metasurface (see sections S2.1.2 and S5.3).

### EO intensity modulation at the threshold

Beyond frequency control, EO driving of the metasurface also enables modulating the laser intensity when operated near the threshold. This effect arises because dynamically shifting the resonance creates a time-dependent detuning between the laser gain and the feedback provided by the metasurface at a fixed optical frequency. At threshold, gain is just sufficient to balance for losses for a single mode $g_{\text{th}}=-\frac{\text{ln}\left[r_{1}r_{\text{ext}}\left(\nu_{\text{res}}\right)\right]}{2L_{i}}$, so the transient detuning can temporarily alter this balance—effectively turning on and off the laser diode and introducing an intensity modulation. Above threshold, where the gains substantially overcome losses, this intensity modulation is expected to be strongly suppressed, in line with our discussion above.

**Fig. 4.**
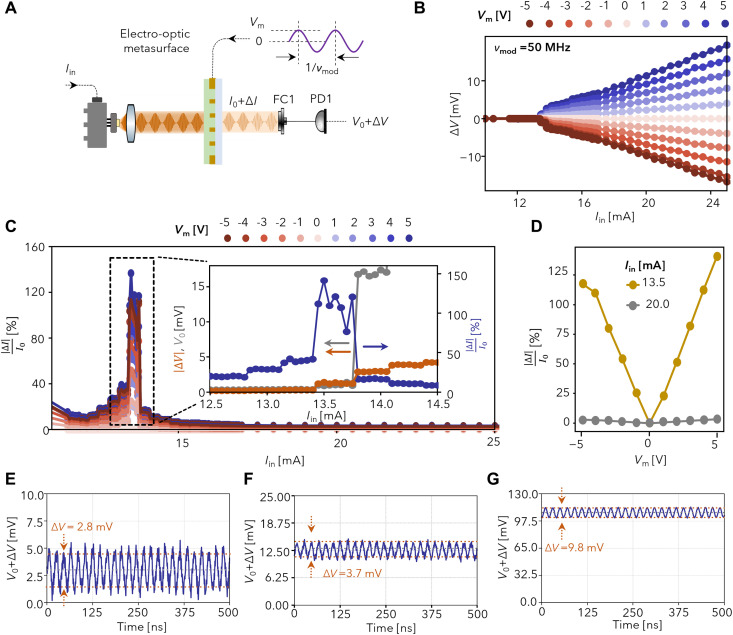
Experimental setup for amplitude modulation. (**A**) An RF voltage with amplitude $V_{m}$ at $\nu_{\text{mod}}=50$ MHz is applied to the metasurface using an arbitrary waveform generator. The lock-in amplifier is referenced to the same frequency $\nu_{\text{mod}}$. After coupling the output light into a fiber using coupler FC1 and directing it to photodiode PD1, the detected signal appears as $V_{0}+\Delta V$, consisting of a dc component $V_{0}$ and an RF component ΔV oscillating at $\nu_{\text{mod}}$. The lock-in amplifier measures the modulated signal ΔV, while the dc component $V_{0}$ is recorded from the dc output of PD1 using a voltmeter. (**B**) The plots show the variation of the detected voltage ΔV as a function of injection current $I_{\text{in}}$ and RF amplitude $V_{m}$, measured at a modulation frequency of $\nu_{\text{mod}}=50$ MHz. The bipolar colorbar indicates the magnitude and sign of ΔV. (**C**) Measured modulation efficiency $\Delta I/I_{0}$ as a function of injection current $I_{\text{in}}$ for various RF amplitudes $V_{m}$ at $\nu_{\text{mod}}=50$ MHz. The efficiency peaks near the laser threshold, exceeding 100% for $V_{m}>4\;V$. Inset: Zoomed-in view showing the absolute voltage variation ΔV (orange), the dc component $V_{0}$ (gray), and the corresponding modulation efficiency $\Delta I/I_{0}$ (blue, right axis) at $V_{m}=5$ V. Mode-hopping events are also visible as discrete jumps in the traces. (**D**) The maximum modulation efficiency is plotted for two regimes: injection currents near threshold $I_{1}=5$ mA (gold) and well above threshold $I_{3}=20$ mA (gray). (**E** to **G**) Oscilloscope time-trace measurements showing $V_{0}+\Delta V$ at fixed voltage ($V_{m}=5$ V) and varying currents. Traces are shown near threshold at $I_{\text{in}}=13.8$ mA, slightly above threshold at $I_{\text{in}}=14.2$ mA, and well above threshold at $I_{\text{in}}=20$ mA.

To demonstrate amplitude modulation, we operate the external cavity system discussed above close to its threshold and monitor the output power using the setup schematically illustrated in Fig. 4A. A detailed view of the experimental configuration is provided in fig. S5. A sinusoidal RF signal with amplitude $V_{m}$ and frequency $\nu_{\text{mod}}=50$ MHz is applied to the metasurface, while the laser output intensity $I_{0}+\Delta I$ is detected by a photodiode yielding an electrical signal $V_{0}+\Delta V$, where $V_{0}$ is the dc component and ΔV is the RF component oscillating at the modulation frequency $\nu_{\text{mod}}$. When sweeping the current from below to above threshold, we find that the absolute intensity modulation is increasing, exhibiting a symmetric response for positive and negative drive voltages (Fig. 4B). However, in many applications, the relative intensity modulation is of highest relevance. Consequently, we quantify the modulation efficiency $\Delta I/I_{0}=\Delta V/V_{0}$ for various injection currents $I_{\text{in}}$ and RF amplitudes $V_{m}$ and find that the efficiency peaks sharply near the lasing threshold and exceeds 100% for drive amplitudes higher than 4 V (Fig. 4, C and D). For background on the threshold behavior, see fig. S15. This highlights the enhanced susceptibility of the intracavity dynamics to reflection modulation when the laser is operating close to threshold. The inset provides a zoomed-in view of the region near threshold for $V_{m}=5\;V$, where the absolute variation ΔV (orange), the dc voltage $V_{0}$ (gray), and their ratio $\Delta I/I_{0}$ (blue, right axis) are shown simultaneously. Discrete steps in all traces indicate mode-hopping behavior as the injection current is varied. When sweeping the RF amplitude from −5 V to +5 V, we find that the absolute intensity modulation increases approximately linearly with $V_{m}$ (yellow curve in Fig. 4D), as expected for the EO effect. Above threshold, the intensity modulation is considerably lower and remains below 5% (gray curve in Fig. 4D). This is confirmed by directly observing the temporal behavior of the amplitude-modulated laser through recording oscilloscope time traces of the photodetected signal at different injection currents (Fig. 4, E to G). The three panels correspond to operation near threshold at $I_{\text{in}}=13.8$ mA, showcasing how the laser is turning on and off (Fig. 4E), slightly above threshold at $I_{\text{in}}=14.2$ mA (Fig. 4F), and well above threshold at $I_{\text{in}}=20$ mA (Fig. 4G). As the injection current increases, the baseline dc component $V_{0}$ grows markedly, while the amplitude of the modulation ΔV increases more slowly, leading to a reduced relative modulation depth in the above-threshold regime. Last, we show that this increased intensity modulation around laser threshold can be reproduced by solving the rate equations (see section S4.3). Since this approach solves for steady-state solutions, it is applicable for the case of slow modulation discussed here, where all laser dynamics occur on substantially faster timescales. We also note that optical powers on the order of ∼5 mW incident on the metasurface may lead to irreversible reduction of the EO activity due to oxygen-induced photodegradation of the chromophores (*77*). In the present work, the optical power incident on the metasurface during laser operation remained well below this reported threshold. No degradation of the resonance position, quality factor, or modulation efficiency was observed over the duration of the experiments (see section S2.1.3).

Overall, these results demonstrate that EO driving of the metasurface-feedback laser enables efficient amplitude modulation around threshold, while keeping intensity modulation small above threshold. In this regime, the laser enables efficient frequency tunability.

## DISCUSSION

This work presents the first integration of a resonant EO metasurface into an ECL, enabling high-speed control of laser emission through bias voltage. Compared to previous designs requiring multiple bulky components, our platform overcomes the challenges in alignment, footprint, and frequency excursion by condensing both the grating and the EO tuning element into a single ultrathin device, effectively reducing the external cavity length.

Using high-*Q* resonances in subwavelength thick metasurfaces, we achieve single-frequency feedback across a bandwidth that exceeds 100 nm (>13 THz). This approach stands in stark contrast with alternative approaches that rely on ring resonators as the feedback medium, which operate on high modal numbers and support modes with typical free-spectral ranges of hundreds of gigahertz to 1 THz, making them more vulnerable to mode hopping or requiring Vernier filtering (*78*). By carefully designing the properties of the optical resonance such as its resonance frequency and *Q* factor, we achieve single-mode operation across a large current range in a compact design. By choosing an in-plane periodically poled film of highly nonlinear molecules JRD1, we achieve efficient modulation of the metasurface refractive index, enabling frequency excursions exceeding 100 MHz and amplitude modulation above 100% around the threshold. We find that the frequency modulation remains stable across a wide range of injection currents $I_{\text{in}}$, while the amplitude modulation efficiency exhibits a pronounced peak near the lasing threshold. Together, these findings establish EO metasurfaces as a powerful platform for active control of laser dynamics in both frequency and amplitude domains.

We envision several future improvements to effectively address shortcomings in our current implementation regarding (i) optical power handling and long-term stability of the poled EO polymer at elevated intensities, (ii) slow environmental drifts of the free-space external cavity at low modulation frequencies, and (iii) tuning range and efficiency constraints set by resonance dispersion, cavity coupling, and passive losses.

For applications requiring higher optical powers, several strategies could further improve device robustness. Improved environmental and material stability, for example through optimized substrates or encapsulation, may increase the sustainable optical power. Encapsulation with dielectric claddings (e.g., SiN) or hermetic sealing in nitrogen atmospheres has been shown to improve the stability of EO polymer devices (*77*). In addition, cross-linkable EO polymers such as HLD1 and HLD2 (*65*) represent promising candidates due to their improved resistance to photochemical and field-induced degradation while retaining the processability required for metasurface integration. In parallel, power-robust inorganic platforms such as lithium-niobate–based EO metasurfaces could be considered, albeit with trade-offs in achievable *Q*-factor and effective EO efficiency due to lower EO coefficients and fabrication constraints in hard crystalline materials.

For applications requiring narrower linewidth lasers, improved cavity designs with more robust optomechanics or active laser stabilization (*79*–*82*) directly via the proposed EO metasurface may be envisioned for improving both the short- and long-term stability.

Last, our design guidelines showcase several avenues for custom-tailoring and improving the frequency excursion of future metasurface-based external cavity lasers. First, metasurface designs relying on quasi-bound states in the continuum (*83*), Mie resonances (*84*), or photonic crystal structures have been shown to achieve narrow linewidths and high reflectivity, opening the possibility to further improve the frequency modulation, laser threshold, and SMSR. Furthermore, introducing controlled structural asymmetry may provide a route to access quasi-bound states in the continuum regimes and further enhance the quality factor within a similar platform as presented in our work (*84*). Second, placing the metasurface closer to the output facet of the laser diode would reduce the cavity length and therefore increase the maximal frequency excursion. Owing to the simplicity of fabrication of our proposed devices, they could potentially be embedded directly onto the laser facet. Third, the nonlinear material also offers remarkable flexibility. On one side, other material platforms could be envisioned in the future, such as lithium niobate (*62*, *85*), hybrid silicon-organic (*86*), III-V materials (*61*), or phase change materials (*87*–*89*). On the other side, the EO material can be poled using electrodes of arbitrary shape, enabling custom-tailoring of their crystallographic properties and, consequently, their EO properties in the 2D plane of the metasurface. This could enable high-speed on-demand generation of laser modes with nontrivial spatial distribution such as those carrying orbital angular momentum (*52*, *53*). Last, the overall efficiency can be improved by minimizing parasitic effects (e.g., suppression of substrate etalon effects via wedged substrates or backside antireflection coatings).

Within the same platform, angular tuning can induce a splitting of the resonance through symmetry breaking. This could enable a more flexible operation, allowing a single device to access multiple lasing frequencies rather than requiring distinct designs, as described in section S5.1.

We anticipate that the design guidelines and modeling we provide will help enhance the performance of these metasurface-controlled external cavity lasers. Their compact form factor, simple fabrication, frequency robustness, and low power consumption are essential characteristics for establishing electro-optically tunable external cavities as a powerful platform for communication and light detection and ranging systems (*1*, *2*, *26*). In imaging systems such as microscopy, their high-speed tunability may provide opportunities to perform in situ spectroscopy by tuning across resonances on timescales faster than molecular dynamics.

## MATERIALS AND METHODS

### Device fabrication and EO activation

The EO metasurfaces were fabricated on a 1-mm-thick quartz substrate using a standard lift-off process (PMMA-MMA) to define a 50 nm–10 nm gold-titanium (Au-Ti) layer. Subsequently, a solution of the nonlinear material JRD1 mixed with PMMA was prepared for spin-coating onto the substrate. This mixture, consisting of 50 wt % JRD1 powder and PMMA in solid form, was blended with cyclopentanone (10 wt %) as a solvent for 12 hours using a swirling machine. The resulting solution was filtered through 0.2-μm polytetrafluoroethylene filters and applied to the chip via a three-step spin-coating process: 500 rpm for 5 s, 1500 rpm for 30 s, and 1000 rpm for 30 s. After spin-coating, the film was hard-baked at 65°C for 24 hours. A uniform layer thickness of $t_{\text{JRD}1}=1.8$ μm was measured after baking. The device was then electro-optically activated through a poling procedure. During poling, an electric field of up to 100 V/μm was applied via the electrodes to the polymeric material under a nitrogen atmosphere, at the poling temperature ($T_{p}=84$°C), for 15 min to allow molecular alignment with the applied field. The electric field was maintained while the sample cooled down to room temperature. A comparison of *I*-*V* curves obtained with a dc source (Keithley 2400) before and after poling shows increased conductivity, as presented in the fig. S1.

In addition, a change in the film’s topography after poling, measured via atomic force microscopy (see fig. S1) confirms that an electric field was successfully applied to the film during poling. As a result of the applied field, the film typically exhibits a mechanical and morphological surface modification. This observation is consistent with reports about electrostatic stress-driven structural reorganization known to occur in polymer thin films (*90*–*94*). After this preliminary inspection, the successful EO activation of the nonlinear chromophores is independently confirmed by quantifying the EO response of the metasurface in a reflection/transmission setup, as described in section S2.1.1.

### EO frequency tuning

To derive the EO-induced shift in lasing frequency, we start from the resonance condition in Eq. 1, which determines the allowed mode frequencies as $\nu_{m}=\frac{\left(m-\frac{\varphi_{0}}{2\pi }\right)c_{0}}{2\left[\left(n_{c}-1\right)L_{i}+L_{\text{ext}}\right]}$. When an EO voltage $V_{m}$ is applied, the metasurface reflection phase shifts from $\varphi_{0}$ to $\varphi_{0}+\Delta \varphi_{\text{eo}}$, altering the round-trip phase condition and thus the lasing frequency from $\nu_{0}$ to $\nu_{0}+\Delta \nu_{m}$. Assuming that the reflection phase φ varies smoothly around the resonance, we linearize its frequency dependence using a first-order Taylor expansion ${\Delta \varphi_{\text{eo}}\approx \frac{d\varphi }{d\nu_{\text{res}}}|}_{\nu_{\text{res}}}\Delta \nu_{\text{res}}$, where ${\frac{d\varphi }{d\nu_{\text{res}}}|}_{\nu_{\text{res}}}$ is the local slope of the phase spectrum at the lasing frequency (where $r_{\text{ext}}$ is maximal) and $\Delta \nu_{\text{res}}$ the shift in the resonance frequency of the metasurface upon applied bias. Inserting this into the round-trip condition gives

$${\Delta \nu_{m,V=V_{m}}\approx \frac{c_{0}}{4\pi \left[\left(n_{c}-1\right)L_{i}+L_{\text{ext}}\right]}\frac{d\varphi }{d\nu_{\text{res}}}|}_{\nu_{\text{res}}}\Delta \nu_{\text{res}}$$ (5)

The resonance frequency shift is related to the EO-induced index modulation by $\Delta \nu_{\text{res}}=\frac{\nu_{\text{res}}\Delta n_{\text{eo}}}{n_{\text{nlm}}}\Gamma_{c}$, yielding

$$\begin{array}{c} \Delta \nu_{m,V=V_{m}}=\frac{c_{0}}{4\pi \left[\left(n_{c}-1\right)L_{i}+L_{\text{ext}}\right]}\frac{d\varphi }{d\nu_{\text{res}}}{|}_{\nu_{\text{res}}}\frac{\nu_{\text{res}}\Delta n_{\text{eo}}}{n_{\text{nlm}}}\Gamma_{c}\\ =\frac{c_{0}}{4\pi \left[\left(n_{c}-1\right)L_{i}+L_{\text{ext}}\right]}\frac{d\varphi }{d\nu_{\text{res}}}{|}_{\nu_{\text{res}}}g_{\text{eo}}V_{m} \end{array}$$ (6)

which corresponds to Eq. 4.

Starting from this formula, we recognize the ingredients that are necessary to achieve a large frequency excursion $\Delta \nu_{\text{pp}}$: a steep slope ${\frac{d\varphi }{d\nu_{\text{res}}}|}_{\nu_{\text{res}}}$ at the lasing frequency, a large EO coupling rate $g_{\text{eo}}$, and a short cavity, corresponding to a small $L_{\text{ext}}$. A steeper phase slope $\frac{d\varphi }{\mathrm{d\nu}}$ corresponds to an increased group delay $\tau_{g}=\frac{1}{2\pi }\frac{d\varphi }{\mathrm{d\nu}}$ and hence increased photon lifetime τ. Since the group delay is an approximate measure of this photon lifetime in Lorentzian resonators, this is directly related to the quality factor by $Q=2\pi \nu_{\text{res}}\tau$. More generally, the group delay is bounded by the photon lifetime, i.e., $\tau_{g}\leq \tau =Q/2\pi \nu_{\text{res}}$ (*95*), with equality approached only in ideal cases. Consequently, a higher quality-factor resonance translates into a linearly increased frequency excursion for a given RF modulation voltage $V_{m}$. The influence of geometric parameters on the quality factor, along with possible optimization strategies, is discussed in section S3.3 (see fig. S8).

### Optical setups and measurements

Characterization of the metasurface resonance was performed using the reflection/transmission measurement setup depicted in the fig. S2. The wavelength of a continuous-wave (CW) laser (Keysight 8164B) was swept over its full available range, from 1490 to 1630 nm, with a resolution around 10 pm. To characterize the resonance of the metasurface, transmission and reflection measurements were conducted using a wavelength-calibrated power meter (Keysight 81624B). The same setup was then used to assess the reflection and transmission modulation strength for both polarization states. An RF voltage with amplitude $V_{m}=5$ V and modulation frequency $\nu_{m}=50$ MHz was generated using an arbitrary waveform generator (Zurich Instruments HDAWG) and sent simultaneously to the device and to a lock-in amplifier (Zurich Instruments UHFLI) for synchronization. The modulation efficiency, expressed as $\eta_{R}=\frac{\Delta I_{R}}{I_{\text{max}}}$, was measured for different laser emission wavelengths.

An antireflection-coated laser diode (SAL-1550-100) is used as a coherent light source, controlled by the ITC4001 TEC module, which provides the required injection current and maintains a constant temperature of the laser diode. An isolator is placed after the EO metasurface to prevent parasitic reflections from returning to the laser diode. A balanced photodiode (PD1, Thorlabs PDB480C-AC) with a 1.6-GHz bandwidth collects the light for the homodyne detection path. The RF output of the photodiode is connected to the UHFLI lock-in amplifier, while the dc output is connected to a Keithley 2400 Source Meter. All power-current (PI) curves presented throughout this work were measured using the dc output of PD1 and read via the Keithley meter. The coupling efficiency of FC1, the splitting ratio of the beam splitter, and the transimpedance gain of the photodiode are all taken into account to calculate the absolute power after the device. For spectral shaping measurements (Fig. 2D), an optical spectrum analyzer (Anritsu MS9740B) was used to measure the emission spectrum. For the zoom-in inset of Fig. 2D, high-resolution measurements were performed using the Apex AP2043B optical spectrum analyzer. The arbitrary waveform generator was used to modulate the device and to provide the reference signal for the lock-in amplifier. The laser light co-propagates in a 50/50 fiber splitter with a stable CW laser (Keysight N7776C). The output of the splitter is sent to a fast photodiode with 5-GHz bandwidth (PD2, Thorlabs DET08CFC). The CW laser is swept over a range of ±200 pm around the lasing mode of the laser diode. An Extra604A Keysight oscilloscope records the output of PD2, and the time trace on the oscilloscope is used as the raw data for spectral analysis.

## Supplementary Materials

### This PDF file includes:

Supplementary Text Figs. S1 to S19 References
