# Supplementary material for: Metasurface-controlled high-speed tunable external cavity lasers
Source: Sci Adv. 2026 Aug 7;12(32):eaee1791. doi: 10.1126/sciadv.aee1791 (PMC13450220; doi:10.1126/sciadv.aee1791)
Supplement: Supplementary file 1 [file sciadv.aee1791_sm.pdf]

Science Advances
AAAS

Supplementary Materials for

Metasurface-controlled high-speed tunable external cavity lasers

Zahra Basiri *et al.*

Corresponding author: Ileana-Cristina Benea-Chelmus, cristina.benea@epfl.ch

Sci. Adv. **12**, eaee1791 (2026)
DOI: 10.1126/sciadv.aee1791

This PDF file includes:

Supplementary Text
Figs. S1 to S19
References

1 Sample details

1.1 Poling of the nonlinear material

To perform the poling procedure, first, the chip is placed in nitrogen atmosphere and is electrically connected to DC probes in a temperature-controlled environment. The nitrogen purge stays constant during poling. After, we apply gradually increasing electric field up to 100 V μm^{-1}. After the applied electric field reached 100 Vμm^{-1}, we start to increase the temperature to a poling temperature of $T = 84°C$ at a rate of $\approx$ 20°Cmin^{-1}. The poling temperature should be optimized for different substrates and nano-photonic architectures, since they have different thermal conductivities and heat distributions during poling, which could require adjustments to the poling schedules. After reaching the required poling field and poling temperature, poling takes place. To ensure homogeneous and complete poling of the molecules, the chip is held at the temperature T = 84°C for 5-10 minutes. A current rise may be observed as a sign of poling. At the end, the heater is turned off, and the chip starts to cool gradually to near room temperature. The poling field should be on until the sample reaches at least 40°C. Fig. S1A-B shows the IV and IVT measurements before and after poling. For freshly spin-coated samples, an increase in the current may be observed after poling, which is an indication of poling. Another indication of the poling is the change in the topography of the polymer. One way to verify this is by measuring the height of the film by Atomic Force Microscope (AFM). For our geometry, poling leads to a periodic topography that is aligned with the interdigitated array of electrodes, as visualized in Fig. S1C-D (two-dimensional map and cut-lines, respectively).

The observed modification of the surface topography after poling reflects a field-induced mechanical response of the polymer film (*92–96*). Under the applied electric field and elevated temperature, the polymer undergoes viscoelastic deformation driven by electrostatic (Maxwell) stresses and internal field-induced forces, leading to nanoscale surface redistribution. Therefore, the AFM measurements primarily confirm that the film responded to the applied poling field, indicating that the biasing conditions were effectively established. The electro optic activation is independently verified through the measured electro-optic response, as discussed in Sec. 2.1.1.

We note that the poling-induced surface modulation can, in theory, act as a weak diffraction grating. However, the measured modulation depth ($h \sim$ 1–2 nm) is deeply subwavelength compared

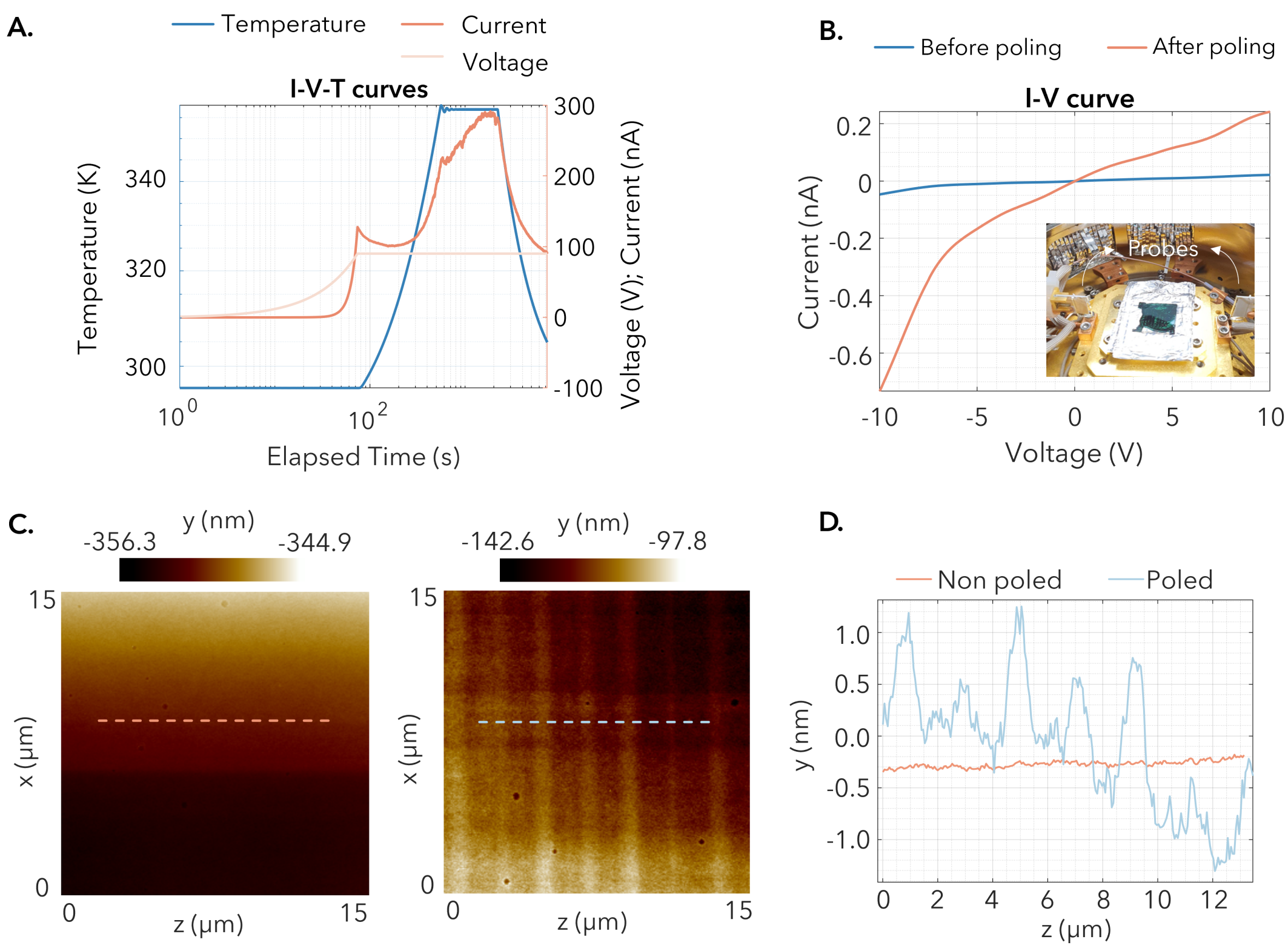

Figure S1: **Nonlinear activation**. **A.** The IVT curves of the poling. During the poling and after reaching the glass transition temperature, the current conducted by the polymer between the two electrodes reaches $\approx$ 300 nA. After tuning off the heater, the current starts to go down. **B.** DC measurement before and after poling. An increase of current is observed after the poling. **C.** & **D.** AFM measurements of the poled and unpoled devices. A change in the topography of the polymer is observed after the poling. Red and blue cut lines show the height of the not-poled and poled devices, respectively.

to the operating wavelength ($\lambda \approx 1550$ nm), resulting in an extremely small phase modulation ($\Delta\varphi \sim 4\pi h/\lambda \ll 1$) and therefore negligible diffraction efficiency (*98*). Consistently, no additional diffraction features or resonances are observed in the reflection spectra over our bandwidth of interest (Fig. 2), indicating that this residual surface modulation does not impact the feedback provided by the metasurface to the laser.

2 Experimental setup

2.1 Device characterization setup

2.1.1 Reflection, Transmission and Modulation measurements

The resonance properties of the electro-optic metasurface were first characterized in a reflection/transmission measurement setup, shown in Fig. S2A. A tunable laser source (TLS, Keysight 8164B) is first linearly polarized to the polarization state of interest via a Half Wave Plate (HWP, Thorlabs WPHSM05-1550) and a Linear Polarizer (LP, Thorlabs LPNIR050). The mirrors M1 and M2 are used to align the light to normal incidence with respect to the device. Prior to the metasurface, the incident light of $2w_0 = 400\ \mu$m is focused onto the device by a first lens (L1) with a focal length of $f_1 = 10$ cm. The transmitted beam is collimated back by a second lens (L2) with a focal length $f_2 = 20$ cm and detected by a photodiode (PD1, Nirvana Auto-Balanced Optical Receiver, Model 2017). The reflected beam from the chip is collimated by the first lens (L1), reflects back from the beamsplitter and is collected by the same photodiode. The red line represents the transmitted light, and the green dashed line represents the reflected light. The measured reflected powers are then normalized to the references to obtain the reflectances that are shown in Fig. S2B for device I with the pitch size of 1.04 μm and device II with the pitch size of 1.06 μm. For the transmission reference, the device is removed, and the transmission is measured with the same components. For the reflectance reference, the device is replaced with a gold mirror (Thorlabs, PF10-03-M01), and the measured reflectance is considered as the reference.

An important property of our metasurfaces is their modulation efficiency, which quantifies the amount of intensity modulation provided by the metasurface in reflection upon an applied bias V_m. The modulation efficiency of the devices, defined as $\eta(\lambda)|_{V_m} = \frac{dI_R(\lambda)}{I_{\max}}|_{V_m}$, is then characterized with the same setup, to also confirm the nonlinear activation of the devices. I_{max} is the reflected intensity at the maximum of the reflectance. For this purpose, the output of PD1 and PD2 is connected to a lock-in amplifier (Zurich Instruments UHFLI), allowing characterisation in both transmission and reflection. An Arbitrary Wave Generator (Zurich Instruments HDAWG) applies the RF modulation of frequency ν_{mod} to the device and sends the signal with the same frequency as the reference to the lock-in amplifier. The TLS sweeps the wavelength and the modu-

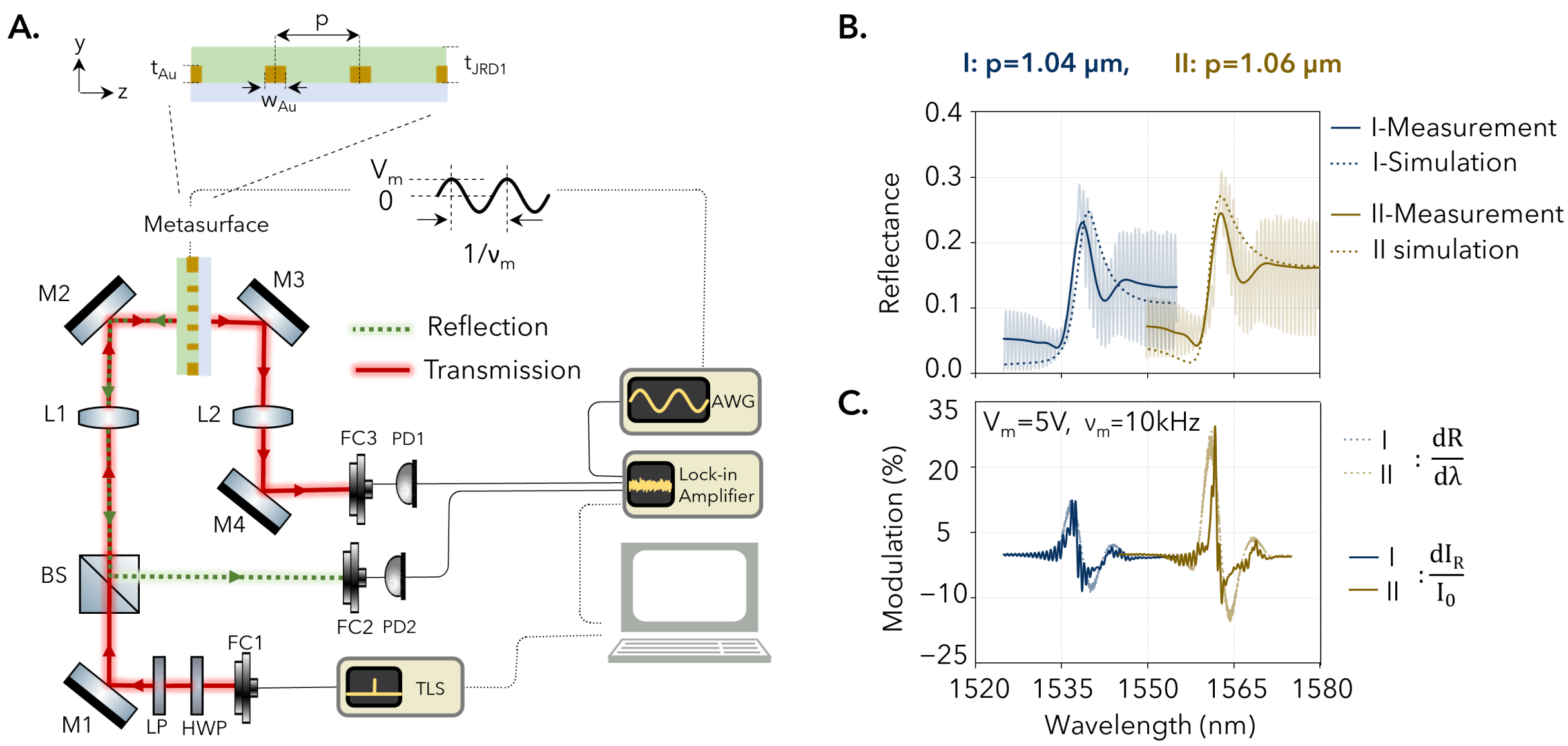

Figure S2: **Device characterization. A.** Schematic of the experimental setup. Red lines indicate the transmission path, and green lines represent the reflection path. The metasurface consists of a periodic array of gold electrodes with pitch size p, thickness $t_{\mathrm{Au}} = 50$ nm, and width $w_{\mathrm{Au}} = 200$ nm, deposited on a nonlinear material with thickness $t_{\mathrm{JRD1}} = 1.8$ μm. **B-C**. (B) Measured reflectance spectra of two devices with pitch sizes $p = 1.04$ μm (Device I, blue) and $p = 1.06$ μm (Device II, gold). Fabry–Pérot fringes are retained (shown transparently) for spectral accuracy. (C) Modulation efficiency measured by the lock-in amplifier as a function of wavelength under sinusoidal RF driving ($V_m = 5$ V, $\nu_{mod} = 10$ kHz). The normalized modulation depth dI_R/I_{max} (where I_{max} is the peak reflected intensity) follows the derivative of the measured reflectance $dR/d\lambda$ (dotted lines), confirming maximal modulation efficiency happening when the slope of the reflectance is maximal. Simulated reflectance spectra (dotted lines in (B)) are obtained using CST unit-cell modeling. The substrate is modeled as fused silica, and the refractive index of JRD1 is implemented using a dispersive model provided by the manufacturer. A chromophore concentration of 50% is assumed, matching experimental conditions. TLS: Tunable Laser Source; L1, L2: Lenses; M1–M4: Mirrors; BS: Beam Splitter; LP: Linear Polarizer; HWP: Half-Wave Plate; FC: Fiber Coupler.

lation of the reflected/transmitted light is measured with the lock-in amplifier for each wavelength. Fig. S2C shows the measurement results of the modulation characterization for devices I and II. The modulation is greatest where the slope of the reflectance is maximal. This experiment provides a direct and reliable confirmation of electro-optic activation. A detailed analysis of the modulation behavior before and after poling, including polarity-dependent measurements, is presented in the Supplementary Materials of our prior work (*67*).

2.1.2 Modulation bandwidth

To characterize the electro-optic bandwidth of the metasurface via the setup shown in Fig. S2, we measure the modulation efficiency as a function of the applied RF modulation frequency. The modulation efficiency is defined as the normalized modulation depth extracted from the optical signal while sweeping the RF drive frequency. The measured response is normalized to its low-frequency value to facilitate comparison across frequencies. Figure S3 shows the resulting frequency response of the device. The modulation efficiency remains nearly constant over a broad range of modulation frequencies and gradually decreases at higher frequencies. The -3 dB cutoff frequency is defined as the modulation frequency at which the response drops to $1/\sqrt{2}$ of its low-frequency value. From this measurement we obtain a cutoff frequency of approximately 150 MHz. We note that this value includes the influence of the RF delivery path used in the experiment (signal generator, cables, and connectors). In the present measurements, standard laboratory cabling rather than impedance-matched high-frequency RF components was employed. As a result, the observed cutoff likely reflects limitations of the external electronics and measurement setup and may therefore underestimate the intrinsic electro-optic bandwidth of the metasurface device.

2.1.3 Power sustainability

The electro-optic polymer platform used in this work can experience degradation of its electro-optic activity at sufficiently high optical powers which we attribute to photooxidation (*78*). In the present work, the maximum optical power incident on the metasurface during laser operation was kept below approximately 2.5 mW. To better understand the power handling of the device, we characterize their electro-optic activity by measuring the modulation efficiency as described in Fig. S2 as a function of optical power. These measurements (Fig. S4) show that the electro-

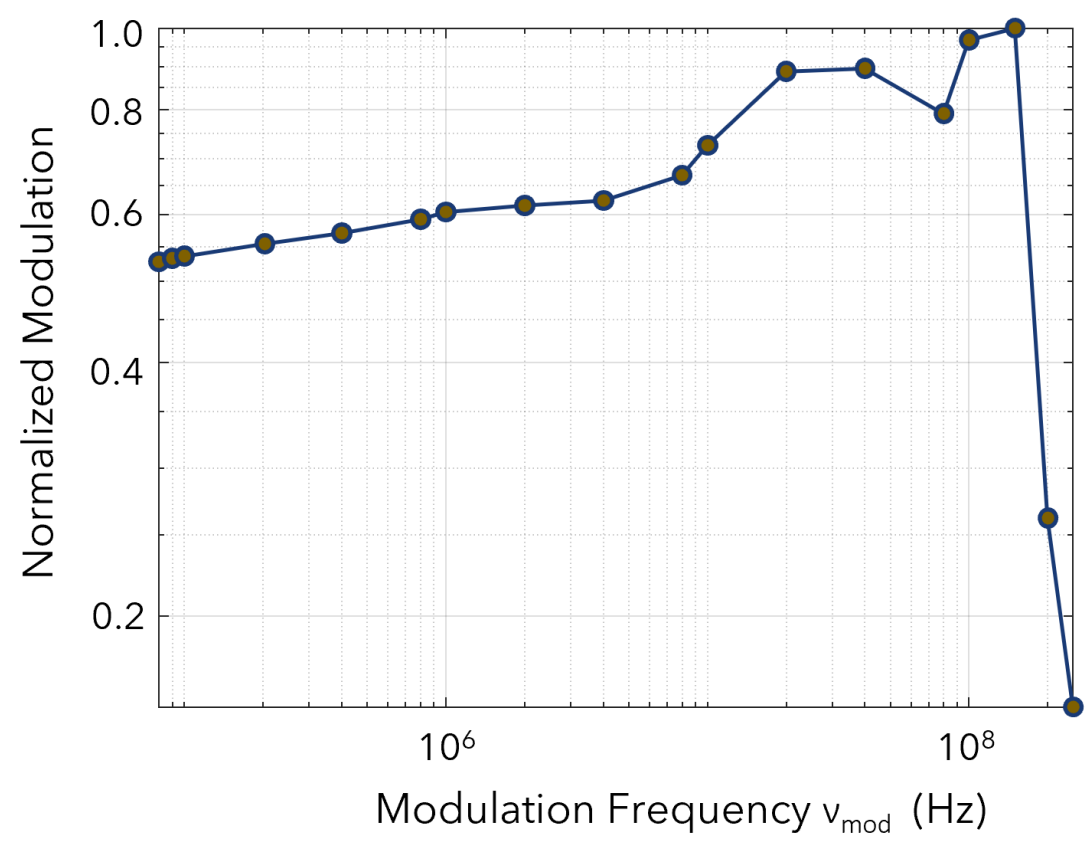

Figure S3: **Normalized modulation efficiency as a function of RF modulation frequency.** The response remains nearly constant over a broad frequency range and exhibits a −3 dB cutoff around ∼ 150 MHz.

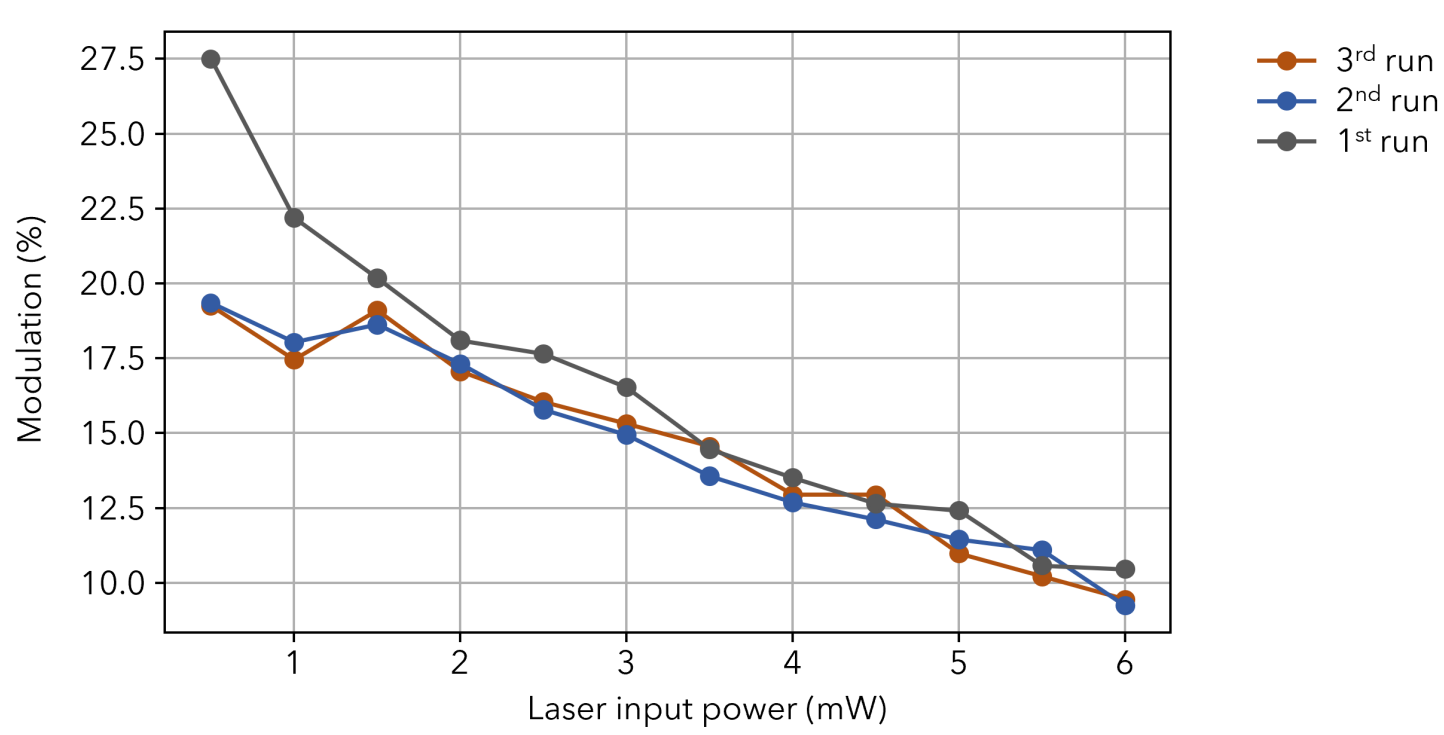

Figure S4: **Measured modulation efficiency as a function of optical power incident on the metasurface.** The measurement was repeated three times to verify reproducibility. A gradual decrease in modulation efficiency is observed with increasing optical power, indicating that the device performs most efficiently at lower optical powers. This trend is consistent with a reduction of the effective electro-optic response, possibly due to partial degradation of the polymer poling at higher optical intensities. However, we observe a recovery of the electro-optic response when returning to low optical powers.

optic response does not abruptly disappear at higher powers. Furthermore, at the power levels used in this work, a maximum modulation efficiency is maintained. Beyond this power level, the modulation depth gradually decreases as the optical power increases. Interestingly, by comparing the modulation efficiency in several consecutive runs, we notice that the electro-optic activity can be (at least partially) recovered by using low optical powers even after applying higher optical powers.

2.2 Frequency and amplitude modulation setup

The setup used for measuring the frequency and amplitude modulation introduced by the metasurface is shown in Fig. S5.

An anti-reflection-coated laser diode (SAL-1550-010) is used as a coherent light source, controlled by the ITC4001 TEC module, which provides the required injection current and maintains a constant temperature of the laser diode. An isolator is placed after the electro-optic metasurface to prevent any parasitic reflections from returning to the laser diode. To achieve optimal alignment of the metasurface with the gain medium, an optical spectrum analyzer (Anritsu MS9740B) is used to measure the emission spectrum below threshold, where the resonance of the metasurface is visible in transmission. For evaluating the sideband suppression when operating single mode we use a high-resolution heterodyne optical spectrum analyzer (Apex AP2043B).

For measuring the intensity modulation, a balanced photodiode (PD1, Thorlabs PDB480C-AC) with a 1.6 GHz bandwidth collects the light for the homodyne lock-in path. An arbitrary waveform generator (HDAWG from Zurich Instuments) was used to modulate the device and to provide the reference signal for the lock-in amplifier. The RF output of the photodiode is connected to the lock-in amplifier (UHFLI from Zurich Instruments), while the DC output is connected to a Source Meter (Keithley 2400). All LI curves presented throughout this work were measured using the DC output of PD1 and read via the Keithley. The coupling efficiency of FC1, the splitting ratio of the beam splitter, and the transimpedance gain of the photodiode are all taken into account to calculate the absolute power after the external cavity.

For measuring the instantaneous frequency, we set up a real-time heterodyne measurement, where a continuous-wave laser (Keysight N7776C) beats with the output of the external cavity laser on a fast photodiode with 5 GHz bandwidth (PD2, Thorlabs DET08CFC). The CW laser is set to a frequency close to the lasing frequency (within a few hundreds of megahertz). A high-bandwidth oscilloscope (Keysight Extra604A) records the output of PD2, and the time trace on the oscilloscope is used as the raw data to calculate the instantaneous frequency as a function of time. In each spectrogram (e.g. shown in Fig. 3 of the main manuscript), the central frequency of the heterodyne signal is given by the wavelength difference between the reference CW laser and the lasing frequency of the device.

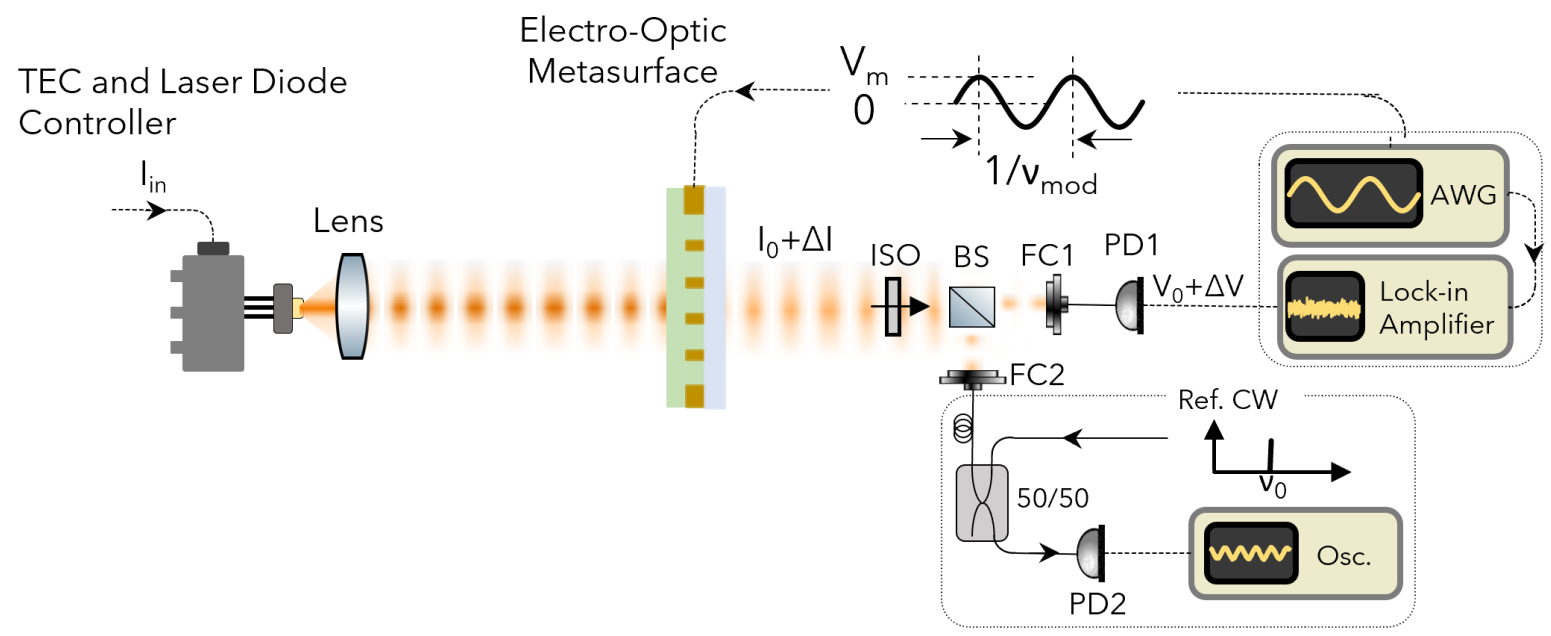

Figure S5: **Measurement Setup**. The output of the laser diode is modulated by the electro-optic metasurface. The transmitted beam is split by a beam splitter into two paths: one directed to the homodyne detection setup for amplitude modulation measurements, and the other to the heterodyne detection configuration for frequency tuning measurements. FC: fiber coupler; BS: beam splitter; CW: continuous wave; AWG: arbitrary waveform generator; PD: photodiode, Osc.: oscilloscope.

3 Metasurface design

In this section, we investigate how variations in the geometrical properties of the metasurface influence its resonant behavior. To this end, we perform frequency-domain numerical simulations using the unit cell module of CST Microwave Studio. We set periodic boundary conditions in-plane and open boundary conditions in propagation direction, both on air side and substrate side. To accurately model the nonlinear response, the dispersive refractive index of a 50% JRD1:PMMA mixture was implemented in CST. A csv file containing this material data is available on the Zenodo repository under https://zenodo.org/uploads/19220102. The electrode pitch p and the nonlinear material thickness t_{JRD1} were used as sweep parameters, while the gold width (w_{Au} = 200 nm) and thickness t_{Au} = 50 nm, also thickness of the nonlinear material t_{jrd1} = 1.8 μm were kept fixed, matching the fabricated devices.

3.1 Optical resonance

In our work, we opt to operate our external cavity on the mode shown in Fig. S6A, which is the first excited guided mode, as visible from the electric field distributions of E_y, E_z. Sweeping the pitch size between p = 1.01–1.07 μm allows to shift the reflectance peak across the telecom band, see r_{ext} in Fig. S6B. Typical maximal reflection coefficients are around r_{max} = 0.25, and the quality factor of these resonances varies between 100 and 250. Finally, around resonance a phase excursion

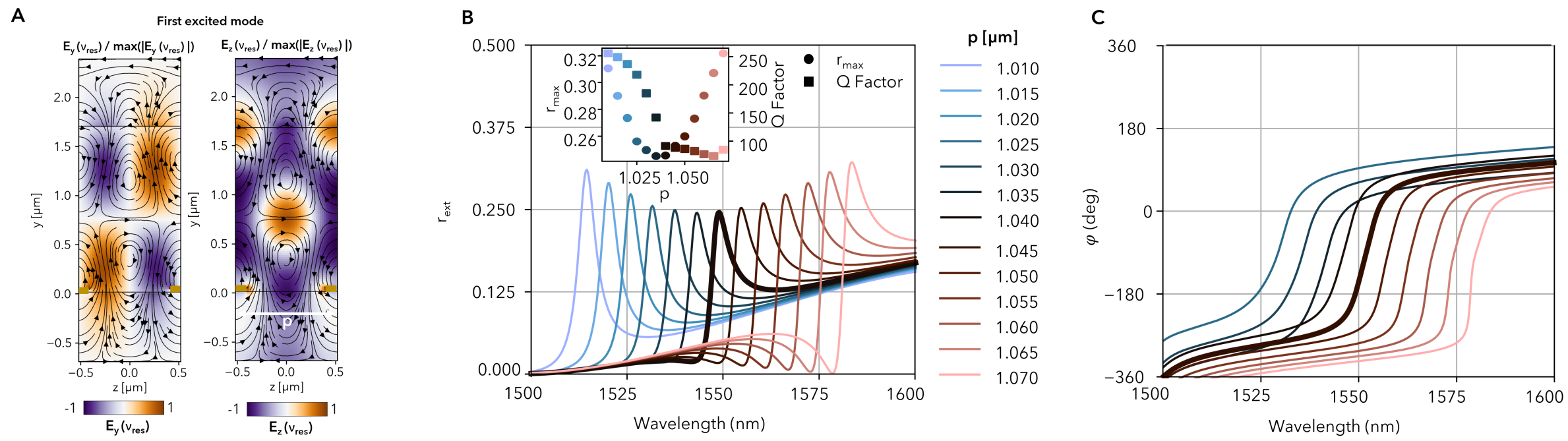

Figure S6: **Impact of electrode pitch size on resonance properties. A**. Simulated field distributions (E_y, E_x) at resonance for $p = 1.02\ \mu$m and $t_{\mathrm{JRD1}} = 1.7\ \mu$m, showing strong confinement within the nonlinear material. **B**. Reflectance spectra for varying pitch sizes ($p = 1.01-1.07\ \mu$m); increasing p leads to a red-shifted resonance and reduced maximum reflectance. Inset: extracted Q-factor and maximum reflectance values. **C**. Phase response of the reflection, centered around zero; larger pitch results in slightly greater phase excursion, enabling stronger phase modulation. Thick line represents the device used in the experiments of the main text.

larger than π is observed. The device used for our external cavity experiments discussed in the main text is shown with a thicker line.

3.2 Overlap calculation

The nonlinear polarization in the orientationally ordered materials, such as polymer JRD1 that we are using in this research, can be described as (*99*)

$$\begin{bmatrix} P_x(\nu_{opt} \pm \nu_{\mathrm{mod}}) \\ P_y(\nu_{opt} \pm \nu_{\mathrm{mod}}) \\ P_z(\nu_{opt} \pm \nu_{\mathrm{mod}}) \end{bmatrix} = \begin{bmatrix} 0 & 0 & 0 & 0 & d_{15} & 0 \\ 0 & 0 & 0 & 0 & 0 & 0 \\ d_{31} & 0 & d_{33} & 0 & 0 & 0 \end{bmatrix} \begin{bmatrix} 2E_x(\nu)E_x(\nu_{\mathrm{mod}}) \\ 2E_y(\nu)E_y(\nu_{\mathrm{mod}}) \\ 2E_z(\nu)E_z(\nu_{\mathrm{mod}}) \\ E_y(\nu)E_z(\nu_{\mathrm{mod}}) + E_z(\nu)E_y(\nu_{\mathrm{mod}}) \\ E_x(\nu)E_z(\nu_{\mathrm{mod}}) + E_z(\nu)E_x(\nu_{\mathrm{mod}}) \\ E_x(\nu)E_y(\nu_{\mathrm{mod}}) + E_y(\nu)E_x(\nu_{\mathrm{mod}}) \end{bmatrix}.$$

Here, P_x, P_y, and P_z are the components of the induced nonlinear polarization at the frequencies $\nu_0 \pm \nu_{\mathrm{mod}}$, and E_x, E_y, and E_z are the corresponding components of the electric field at the optical frequency ν and RF ν_{mod}, interacting in the polymer. The matrix elements d_{15}, d_{31}, and d_{33} represent the nonzero components of the second-order nonlinear susceptibility tensor $d_{ijk} = -\frac{n^4}{4} r_{ijk}$.

To analyze the behavior of the optical and RF fields, one needs to solve the nonlinear wave equations, keeping in mind that the nonlinear coefficient $d_{33}(\mathbf{r})$ is space-dependent in our case, since poling is applied via the interdigitated array:

$$\left[\nabla^2 - \frac{\epsilon_r}{c_0^2}\frac{\partial^2}{\partial t^2}\right] E_z^{\mathrm{nl}}(\mathbf{r},t) = \mu_0 \frac{\partial^2}{\partial t^2}\left(d_{33}(\mathbf{r}) E_z^{\mathrm{opt}}(\mathbf{r},t) E_z^{\mathrm{mod}}(\mathbf{r},t)\right) \tag{S1}$$

with the following definitions of the fields

$$E_z^{\mathrm{nl}}(\mathbf{r},t) = E_0^{\mathrm{nl}}\, e_{opt}(z,y)\, e^{-i(\omega_{\mathrm{nl}} t + \varphi_{\mathrm{nl}}(t))}\, e^{-ik_y^{\mathrm{nl}} y}\, e^{-ik_z^{\mathrm{nl}} z}, \tag{S2}$$

$$E_z^{\mathrm{opt}}(\mathbf{r},t) = E_0^{\mathrm{opt}}\, e_{\mathrm{opt}}(z,y)\, e^{-i(\omega_{opt} t + \varphi_{opt}(t))}\, e^{-ik_y^{opt} y}\, e^{-ik_z^{opt} z}, \tag{S3}$$

$$E_z^{\mathrm{mod}}(\mathbf{r},t) = E_0^{\mathrm{mod}}\, e_{\mathrm{mod}}(z,y)\, e^{-i(\omega_{\mathrm{mod}} t + \varphi_{\mathrm{mod}}(t))}\, e^{-ik_y^{\mathrm{mod}} y}\, e^{-ik_z^{\mathrm{mod}} z}. \tag{S4}$$

Solving this equation as described in (*67*) allows to define the overlap Γ_c as

$$\Gamma_c = \frac{\iint \left|e_{\mathrm{opt}}(z,y)\right|^2 \left|e_{\mathrm{mod}}(z,y)\right|^2 dz\, dy}{\iint \left|e_{\mathrm{opt}}(z,y)\right|^2 dz\, dy}. \tag{S5}$$

describing the vectorial overlap between the normalized optical field $e_z^{\mathrm{opt}}(z,y) = \frac{E_z^{\mathrm{opt}}(z,y)}{E_0^{\mathrm{opt}}}$ and the normalized RF field $e_z^{\mathrm{mod}}(z,y) = \frac{E_z^{\mathrm{mod}}(z,y)}{E_0^{\mathrm{mod}}}$. Here, $E_0^{\mathrm{opt/mod}} = \sqrt{\frac{\iint |E_z^{\mathrm{opt/mod}}(z,y)|^2\, dz\, dy}{S_{\mathrm{eff}}}}$, where $S_{\mathrm{eff}} = \frac{\left(\iint |E_z(z,y)|^2\, dz\, dy\right)^2}{\iint |E_z(z,y)|^4\, dz\, dy}$ is the effective area of the fields. Therefore, Γ_c can be calculated directly from the electric fields $E^{opt}(z,y)$ and $E^{mod}(z,y)$ retrieved from numerical simulations. Fig. S7 shows the simulation results of the calculated electric field, normalized to E_0. The overlap factor obtained for mode this optical mode is $\Gamma_c = 0.14$. The corresponding RF fields were obtained using the electrostatic solver of CST.

3.3 Impact of the geometry on the quality factor

The quality factor of the principal guided-mode resonance (GMR) results from the interplay between radiative leakage and material absorption. In the present device, $Q \sim 200$ reflects a

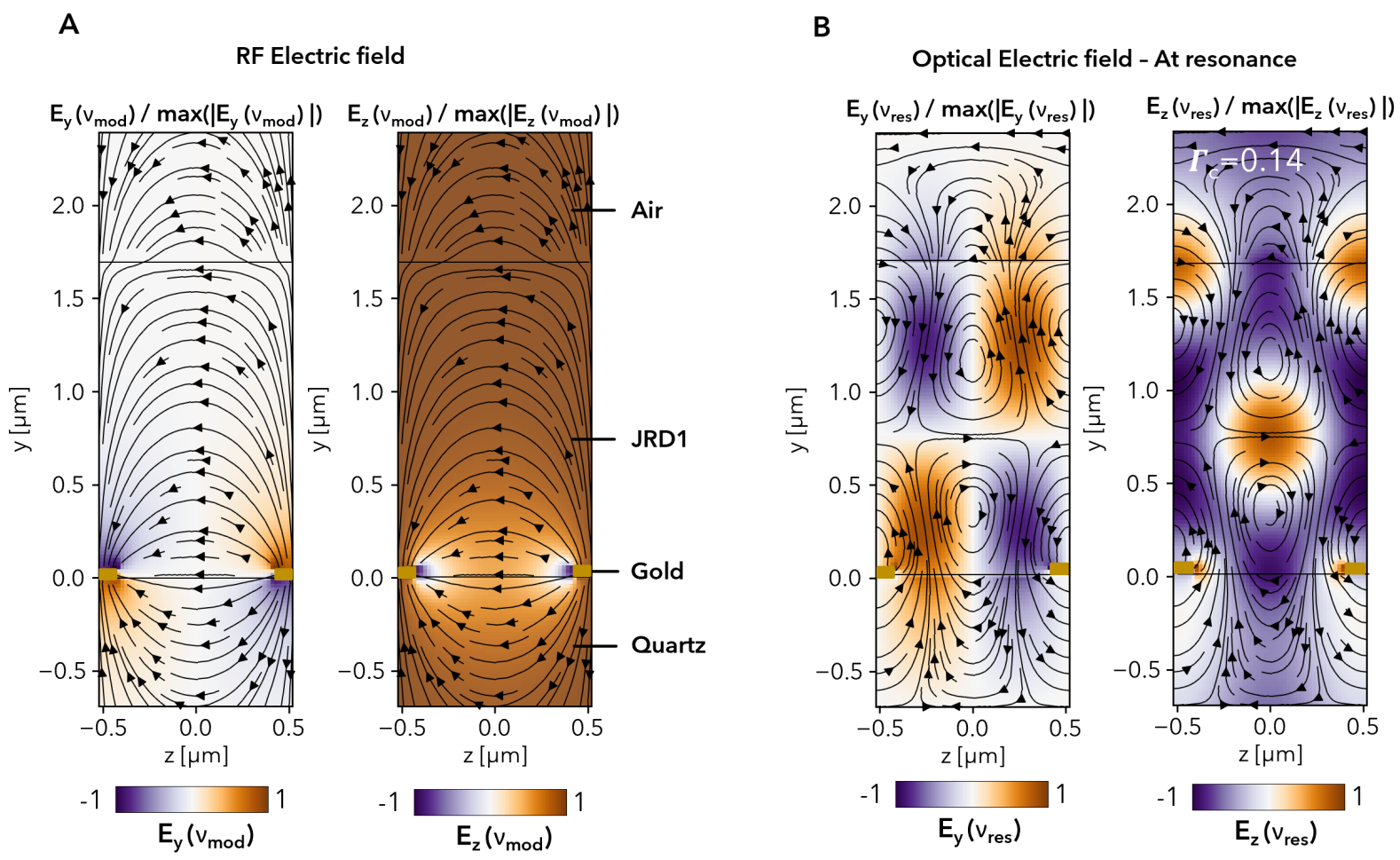

Figure S7: **Calculation of the spatial overlap factor** Γ_c. **A**. RF field applied via the interdigitated array of electrodes. **B.** Optical field on resonance. Both color plots show the normalized field E_z and E_y and their vectorial orientation in-plane. Here, the optical field, the RF field and the poling fields/r_{33} are in the same plane. The calculated overlap is $\Gamma_c = 0.14$.

compromise between sufficient radiative coupling required for efficient external cavity feedback and dissipative losses in the metallic nanostructures and nonlinear polymer layer. To evaluate the potential for increasing Q within the current framework, we performed systematic parameter sweeps of the nonlinear polymer thickness t_{JRD1}, the gold thickness t_{Au}, and the gold stripe width w_{Au} (Fig. S8). The simulations show that Q is highly sensitive to geometric variations and the associated modification of the resonance condition. Varying the polymer thickness t_{JRD1} shifts the GMR wavelength and alters the effective refractive index of the guiding region. For the present operating point ($t_{\mathrm{JRD1}} \approx 1.7\ \mu$m), the device operates in an intermediate regime. Both thinner ($\sim 1.5\ \mu$m) and thicker ($\sim 1.9\ \mu$m) layers can yield higher Q values depending on the resulting phase-matching and coupling conditions. Experimentally, this parameter can be adjusted via the JRD1 concentration and spin-coating process. In contrast, increasing the gold thickness t_{Au} systematically reduces Q, consistent with enhanced ohmic losses in the metal. Thus, simply increasing the metal thickness does not improve resonance performance. The gold stripe width w_{Au} strongly influences both the resonance strength and lineshape. A width of around $w_{\mathrm{Au}} \approx 180$ nm (compared to 200 nm in the current implementation) would provide the highest quality factor in our sweep, although at the expense of lower reflectivity. This is undesirable since it would increase the laser threshold

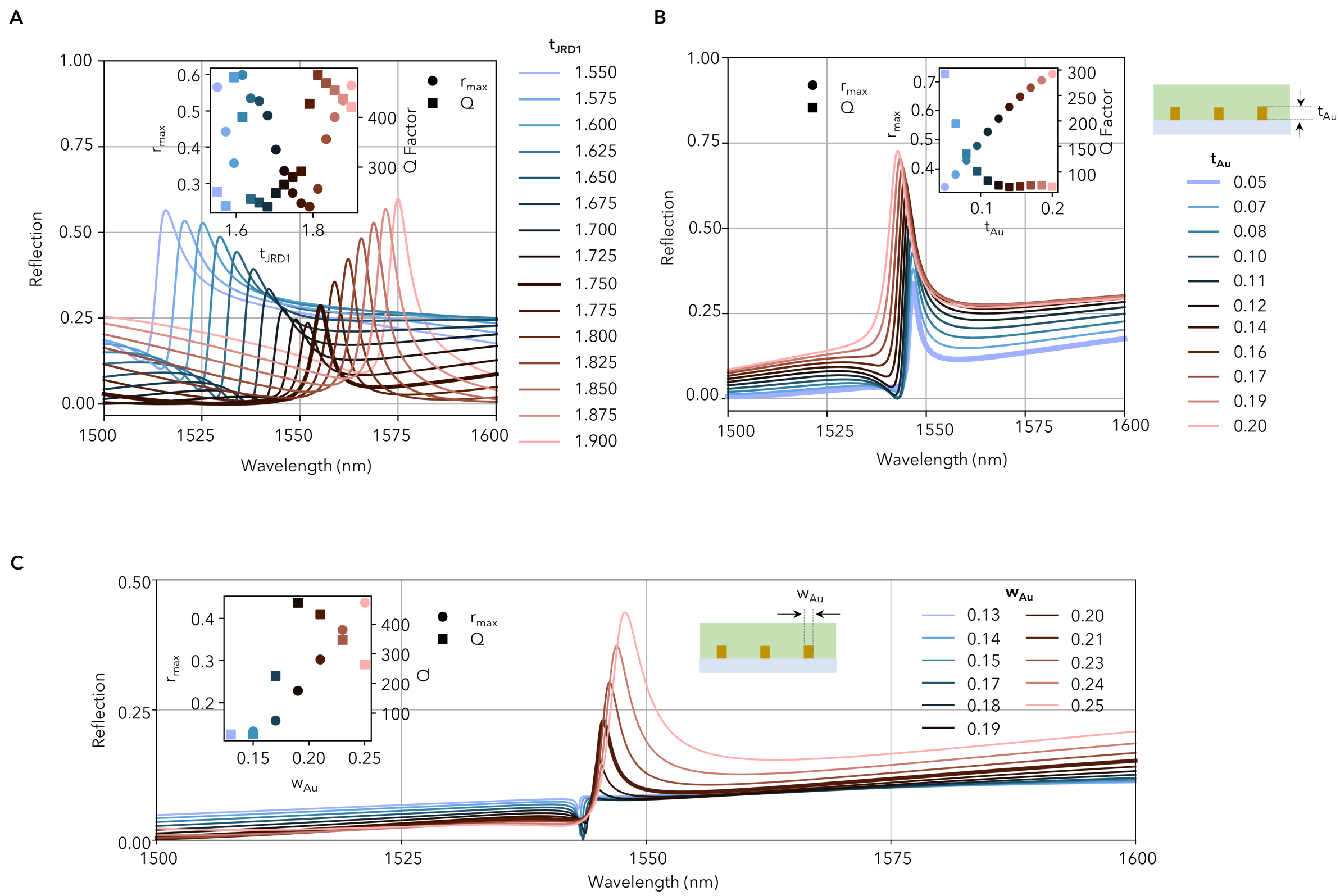

Figure S8: **Simulated impact of geometric parameters on the principal guided-mode resonances. A**. Reflection spectra for varying nonlinear polymer thickness t_{JRD1}. The inset shows the extracted maximum reflectivity r_{max} and corresponding quality factor Q as a function of t_{JRD1}. **B**. Reflection spectra for different gold thicknesses t_{Au}, with inset showing r_{max} and Q. Increasing t_{Au} leads to reduced Q due to enhanced dissipative losses. **C**. Reflection spectra for varying gold stripe width w_{Au}. The inset summarizes r_{max} and Q versus w_{Au}.

current. Overall, these results indicate that moderate geometric optimization can increase the quality factor within the present architecture. However, improvements must be carefully balanced against maintaining a resonance lineshape and reflectivity compatible with robust laser operation.

3.4 Phase modulation across the resonance

Achieving a large frequency and amplitude tuning relies on strong changes in the resonance frequency as a function of applied voltage. As discussed in the main text, maximizing these necessitates resonances with large phase slope around the resonance. Since this voltage directly impacts the refractive index of the JRD1 slab, it is insightful to simulate the resonance of the

metasurface as a function of the refractive index changes in the nonlinear material and analyze its reflection amplitude and phase as a function of an index shift Δn ranging from -0.004 to $+0.004$. As shown in Supplementary Fig. S9, both the reflection amplitude and the associated phase response remain similar across this range, indicating that the resonance properties are robust to small refractive index variations. From the simulations, a resonance shift of approximately 0.26 THz is observed for a total index change of $\Delta n = 8 \times 10^{-3}$ (Fig. S9A and C). By analyzing the phase response, we find its slope at resonance to amount to $\frac{\Delta\varphi}{\Delta\lambda} = 27°/\text{nm}$, which is equivalent to $\frac{\Delta\varphi}{\Delta\nu} = 31°/\text{THz}$ (Fig. S9B).

This phase modulation introduces a frequency modulation of the external cavity laser with a magnitude that depends on the length of the external cavity. In line with formula 4 of the main text, shorter cavities enable larger frequency excursion. We model this behavior for varying external cavity lengths and shifts of the resonant frequency $\Delta\lambda_{res}$, at fixed phase slope of $\frac{\Delta\varphi}{\Delta\lambda} = 27°/\text{nm}$ (Fig. S9D). We find that nearly 11 times larger frequency modulation would be possible by decreasing the cavity length to 0.5 mm, which would correspond to attaching the metasurface directly to the facet of the laser diode.

Finally, the Q-factor of the resonance also plays a major role, since higher Q-factors are associated with steeper phase slopes at resonance. In line with formula 4 of the main text, the total frequency excursion depends linearly on this slope. We model this behavior for various shifts of the resonant wavelength in Fig. S9E. Simultaneously achieving frequency shifts larger than 1 nm and phase slopes larger than $\frac{\Delta\varphi}{\Delta\lambda} = 70°/\text{nm}$ would enable frequency excursions larger than 1 GHz, even for an extended cavity length of $L_{ext} = 2.5$ cm.

4 Modeling of laser behavior

The reflection $r_{ext}(\nu)$ directly impacts photon lifetime $\tau_p(\nu)$, saturation photon number n_{sat}, and effective absorption cross section $\sigma_{eff}(\nu) = \dfrac{\sigma_0 \cdot g(\nu)}{1 + \frac{n_\nu}{n_{sat}}}$. Therefore, changing the properties of the $r_{ext}(\nu)$ directly impacts the number of photons in the cavity at given frequency n_ν and, as a result, P_{out}. To simulate the impact of different resonance properties on the lasing condition, we numerically solve the photon density and population rate equations. We consider the laser a 4 level system, where the lasing occurs between the first and the second levels with N_1 and N_2 as the lower

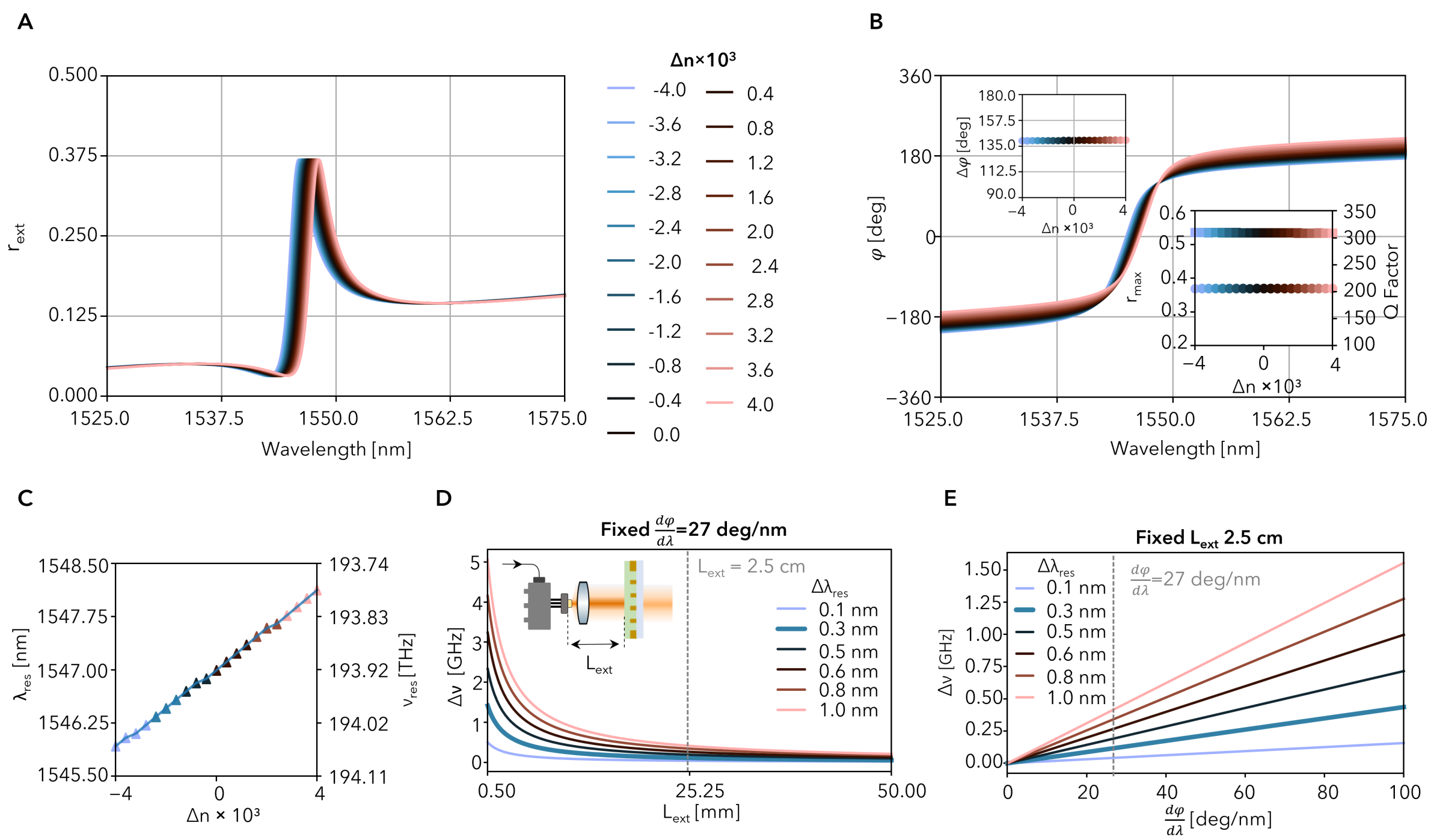

Figure S9: **Impact of the refractive index shift on the resonance properties**. **A**. Reflection amplitude and **B**. Reflection phase for various refractive index changes. The plots are showing the linear simulation of reflection amplitude and phase for various shift of the refractive index of the nonlinear material Δn, starting from -0.004 to 0.004, where the refractive index of the polymer is defined as $n_{\text{eo}} = n_{\text{nlm}} + \Delta n$. It can be seen that the resonance properties do not change dramatically across this change. **C**. Resonance shift due to the refractive index modulation. The shift of $\Delta\nu_{\text{res}} = 0.26$ THz ($\Delta\lambda_{\text{res}} = 1.49$ nm) is occurring for a total shift of $\Delta n = 8 \times 10^{-3}$. Assuming the shift in the resonance frequency with the Δn is happening roughly linearly, this shift can be mapped to the measurements. **D**. Impact of cavity length. For a given electro-optic frequency shift and phase , which is controlled by the applied voltage V_m and the efficiency of the device, and a fixed phase slope, which is detemined by the resonance Q-factor, one can tune the frequency excursion of the laser $\Delta\nu$ by also changing the external cavity length. Based on the Eq. 4 in the main text, the frequency excursion is inversely proportional to the cavity length. Therefore by reducing the cavity length, larger frequency excursions would be achievable. **E**. Impact of the phase slope (Q-factor) on frequency excursion. For a fixed cavity length and fixed resonance shift, the frequency excursion changes linearly with respect to the phase slope.

and upper states populations, and for the sake of simplicity, we neglect the population of N_0 and N_3 levels (*18, 71*).

$$\frac{dn_\nu}{dt} = (N_2 - N_1)\, n_\nu\, \sigma(\nu)c - \frac{n_\nu}{\tau_p} + \sigma(\nu)cN_2 \tag{S6}$$

$$\frac{dN_2}{dt} = \frac{I_{in}}{eV_c} - (N_2 - N_1)\, n_\nu\, \sigma(\nu)c - \frac{N_2}{\tau_{21}} \tag{S7}$$

$$\frac{dN_1}{dt} = (N_2 - N_1)\, n_\nu\, \sigma(\nu)c - \frac{N_1}{\tau_{10}} + \frac{N_2}{\tau_{21}} \tag{S8}$$

The term I_{in} is the injection current supplied to the laser, V_c is the active region volume, and e is the elementary charge. The parameter $\sigma(\nu)$ stands for the emission cross-section at the lasing frequency which we modeled as a Gaussian function $\sigma(\nu) = \sigma_0 \exp\left(-\frac{1}{2}\left(\frac{\nu - \nu_{\text{res}}}{\Delta\nu_{gain}}\right)^2\right)$ where ν_{res} is the center frequency at 1550 nm, and $\Delta\nu_{gain} = 75$ nm is the gain bandwidth. τ_p is the photon lifetime in the cavity, and τ_{21} and τ_{10} are the carrier lifetimes between the respective energy levels. We numerically integrate the coupled rate equations (S6)-(S8) to calculate the unknowns n_ν, N_1, and N_2. The initial conditions for the photon density and carrier populations are set to $[n_\nu, N_2, N_1] = [0, 0, 0]$. The simulation time domain is set to $t_{\text{span}} = (0, 5\,\text{ns})$, with 10000 time points for evaluation, allowing for the numerical integration of the rate equations under realistic laser cavity and gain conditions, including the impact of wavelength-dependent properties on the lasing condition. Finally, we compute the output power $P_{\text{out}}(\nu) = (1 - r_{ext}(\nu))\,\frac{\beta_{\text{sp}} n_\nu h\nu}{\tau_p(\nu)}$ by solving the steady-state rate equations for each wavelength and injection current and finding steady-state photon number n_ν.

We used the following parameters for our calculation: Planck's constant $h = 6.626 \times 10^{-34}$ J · s, the speed of light $c = 3 \times 10^8$ m/s, the elementary charge $e = 1.602 \times 10^{-19}$ C, the carrier lifetimes $\tau_{21} = 0.6$ ps and $\tau_{10} = 0.1$ ps, the spontaneous emission coupling factor $\beta_{\text{sp}} = 10^{-4}$, the confinement factor $\Gamma = 0.3$, the differential gain coefficient $G = 2 \times 10^{-16}\,\text{cm}^2$, and the background carrier density $N_0 = 10^{16}\,\text{cm}^{-3}$. For the laser cavity parameters, we use a constant reflectivity $r_1 = 0.99$ and variable $r_{ext}(\nu)$, a cavity length of $L_c = 0.5$ mm, and a cavity width and height of $w_c = h_c = 2\,\mu$m, yielding a cavity volume of $V_c = w_c \times h_c \times L_c$. We calculate the mirror (radiative) losses using $\alpha_{\text{rad}}(\nu) = -\frac{\ln(r_1 r_{ext}(\nu))}{2L_i}$ and assume the internal losses to be $\alpha_{in} = 7.6\ cm^{-1}$. The total loss coefficient is $\alpha_{\text{tot}}(\nu) = \alpha_{\text{rad}}(\nu) + \alpha_{\text{in}}$, the photon lifetime in the cavity is given by $\tau_p(\nu) = \frac{1}{\alpha_{\text{tot}}(\nu)c}$

and the saturation photon number is $n_{\text{sat}}(\nu) = \dfrac{\tau_p(\nu)}{\Gamma\sigma_0\tau_{21}c}$.

4.1 Resonance wavelength

Based on the rate equations, we now study the impact of the resonance wavelength of the metasurface on laser emission. This allows to investigate how the detuning between the peak frequency of the gain $g(\nu)$ and the peak of the reflection $r_{ext}(\nu)$ can impact the threshold current, as well as the emitted spectrum. For the reflection, we assume a Lorentzian-like profile $r_{ext}(\nu) = \dfrac{1}{\delta\nu_{res} - i(\nu - \nu_{res})}$, where $\nu_{res} = c/\lambda_{res}$ is the resonance frequency of the metasurface and $\delta\nu_{res}$ its linewidth. We assume $\delta\nu_{res} = 10\,\text{nm}$. The normalized magnitude of $r_{ext}(\nu)$ was then scaled to match the desired peak reflectivity $r_{max} = 0.4$: $r_{ext}(\nu) = r_{max}\dfrac{|r_{ext}(\nu)|}{\max(|r_{ext}(\nu)|)}$. We update the corresponding photon lifetime $\tau_p(\nu) = \dfrac{1}{\alpha_{\text{tot}}(\nu)c}$, saturation photon number $n_{\text{sat}}(\nu) = \dfrac{\tau_p(\nu)}{\Gamma\sigma(\nu)\tau_{21}c}$, and effective cross-section $\sigma_{\text{eff}}(\nu) = \dfrac{\sigma(\nu)}{1 + n_\nu/n_{\text{sat}}} = \dfrac{g(\nu)}{(N_2 - N_1)}$ at each frequency.

As a first step, we sweep the values of λ_{res} across the wavelength range of 1400 nm to 1700 nm. Fig. S10A shows the simulation results of the output power of the external cavity when operated above threshold ($I_{in} = 20$ mA). We observe that the output power decreases with increasing detuning. At the same time, the threshold current also increases (Fig. S10B and inset, showing relation between threshold current I_{th} and gain g for different λ_{res}). The kink in the simulated L–I curves in Fig. S10B is a consequence of gain saturation in the model, governed by the saturation photon number n_{sat} (*18, 71*).

This numerical analysis showcases the importance of designing the metasurface such that its resonance aligns with the peak of the gain for achieving low-threshold lasing.

4.2 Peak reflection

The maximum of the reflection r_{max} also impacts the threshold current and the output power of the external cavity. We analyze this behavior by modeling various values of r_{ext} spanning from 0.1 to 0.99, see Fig. S11. As expected, the threshold current is decreasing by increasing the r_{max}. At the time, the output power is decreasing when increasing the r_{max}, since less light is coupled out of the

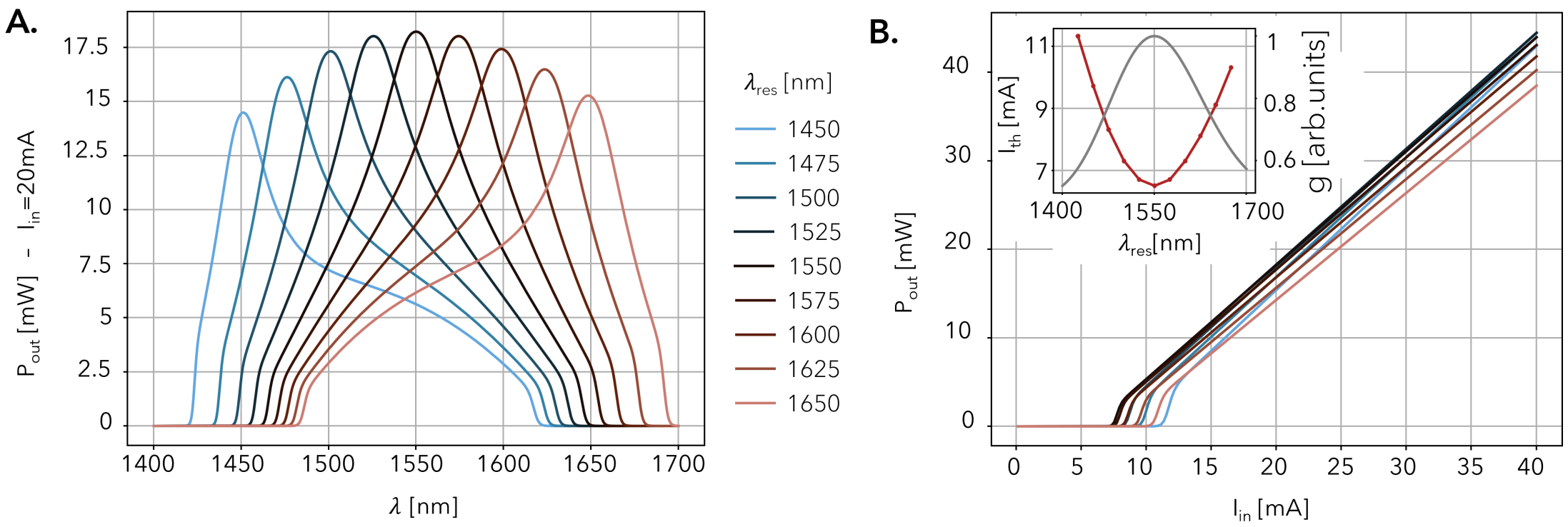

Figure S10: **Solution of the rate equations for different resonances wavelengths of the external cavity**. **A**. Output power P_{out} as a function of the de-tuning between the gain peak and the resonance of the metasurface for I_{in} = 20 mA and r_{max} = 0.4. The output power is decreasing by increasing the de-tuning. **B**. PI-curves and threshold current I_{th} for different λ_{res}. The threshold current is decreasing while decreasing the detuning, and it is minimum for λ_{res} = 1550 nm, where the $g(\nu)$ is maximum.

cavity. We find that our design reflectance of $r_{ext} \approx 0.2$ is a good compromise between the two.

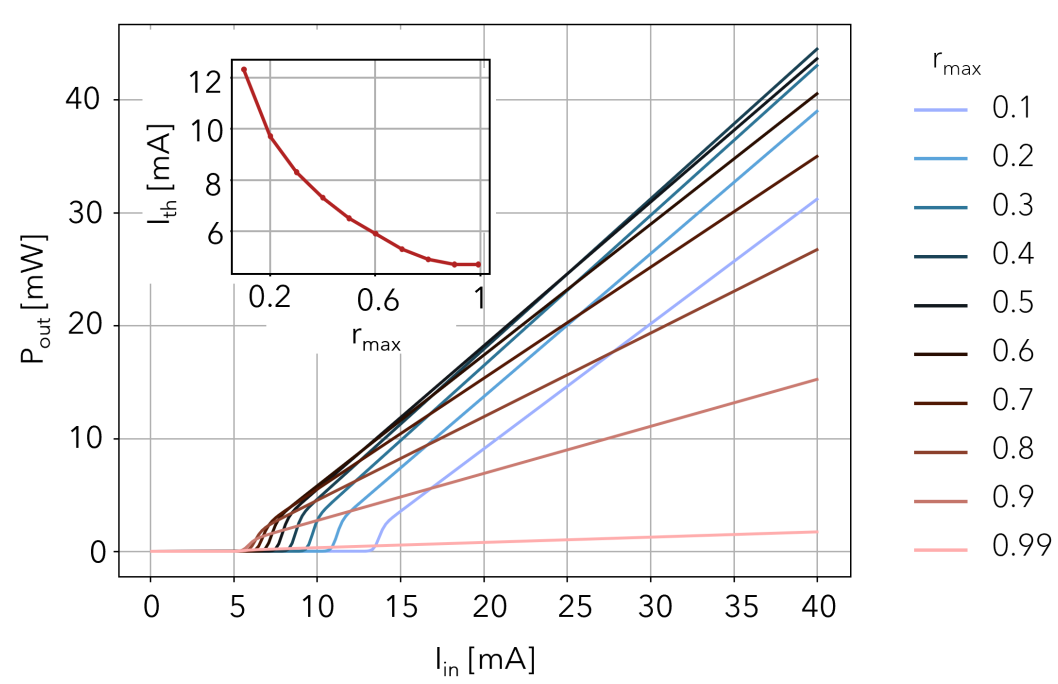

Figure S11: **Solution of the rate equations for different maximum reflectances of the external cavity.** LI-curves and threshold current I_{th} for different r_{max}. The threshold current is decreasing while increasing the r_{max}, and it is minimum for λ_{res} = 1550 nm. The exponential dependence od the I_{th} to r_{max} is highlighted.

4.3 Photon number modulation

In the main text we find that above threshold, the metasurface introduces primarily a frequency tuning, since its modulation results in stronger changes in the phase, rather than its amplitude, of the reflection. This aspect is discussed in Note 3.4. In this section, we complement our analysis by a study where we assume the magnitude of the reflection to be modulated as a function of time as $r_{ext}(t) = r_{max} + \varepsilon \cdot \cos(2\pi\nu_{mod}t)$, where $\epsilon << 1$, and ν_{mod} = 1GHz is the modulation frequency.

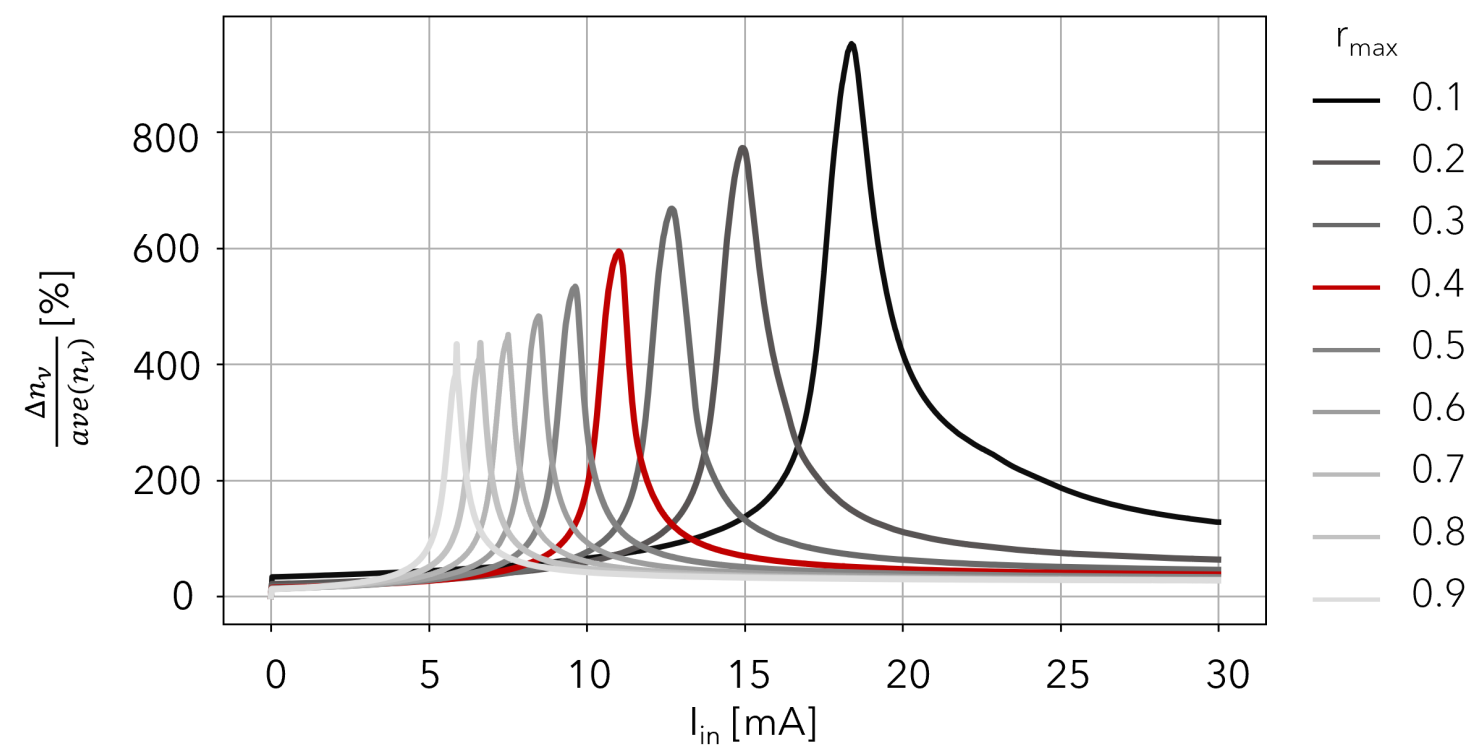

Figure S12: **Modulation of number of photons in the cavity.** The plot shows the modulation of n_ν for different maximum reflection r_{max}. As it was shown before the threshold current decreases with the increase of r_{max}. As a result, the injection current at which the modulation is maximum is also reduced by increasing the r_{max}.

Although we know this modulation to be low (since $\frac{dr}{d\nu}$ is zero at the lasing frequency), we find this analysis to be instructive especially when understanding the increased intensity modulation of the output power around threshold. The output power depends on the intracavity power $P_{in} = \beta h\nu c \frac{n_\nu}{2L_i}$ as $P_{out} = (1 - r_{ext}(\nu)^2)P_{in}$ which is related to $r_{ext}(\nu)$.

We study the impact of a time-varying $r_{ext}(t)$ on the number of photons in the cavity for fixed $\epsilon = 0.05$ and varying r_{max}. We plug the modulated $r_{ext}(t)$ into the rate equations and solve for $n_\nu(t)$. We then calculate the ratio $\eta_{n_\nu} = \frac{\Delta n_\nu}{mean(n_\nu)}$, with Δn_ν being the peak-peak photon number variation. This quantifies the efficiency with which the number of photons in the cavity is modulated. The ratio η_{n_ν} is plotted in Fig. S12A for various r_{max}. The ratio is calculated for injection currents from 0 to 30 mA. It can be seen that the modulation efficiency is maximum around the threshold of each device, consolidating the measurement results shown in Fig. 4 of the main manuscript. By increasing the r_{max} the threshold current is decreasing, however, η_{n_ν} is also decreasing.

The modulation also depends on the variation amplitude ε, which is equivalent to ΔR induced by the RF. To study the impact of this parameter on the modulation of n_ν, we fix r_{max} at 0.4 and sweep ε from 0.01 to 0.09 (Fig. S13).

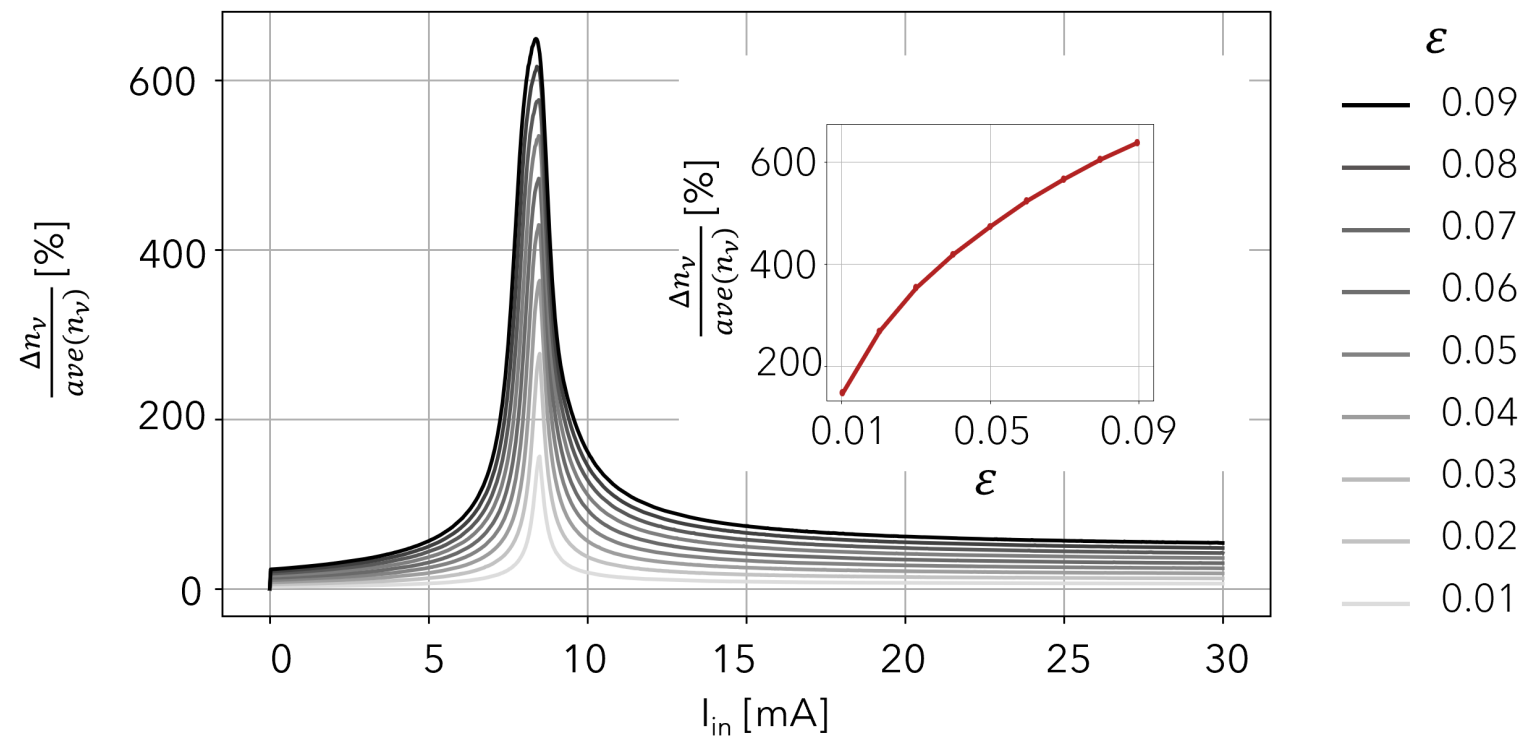

Figure S13: **Intensity modulation for varying reflectances**. ε is swept from 0.01 to 0.09. The relative change in intra-cavity photon number is then calculated from the rate equations for various injection currents I_{in}. The modulation of n_ν is increasing nonlinearly with ε.

5 Supporting measurements

5.1 Spectral shaping

Building on the previous section, where we demonstrated how resonance characteristics can be engineered, in this section we provide further measurements showcasing how they affect the lasing condition. We compare the electrical and spectral characteristics of external-cavity lasers when integrated with metasurfaces of varying resonance wavelength by adapting the pitch size $p = 1.04$ (device I) and 1.06 μm (device II), respectively. Their corresponding reflectances are shown in Fig. S2B(a). When comparing these two devices, we find that device II has a larger threshold and higher slope efficiency (Fig. S14A). This can be explained by the fact that device I, although having a lower maximum reflectance $r_{\max}$, its resonance is less detuned from the maximum of the gain spectrum ν_0, resulting in a lower lasing threshold. Device II, on the other hand, exhibits a higher $r_{\max}$, which would reduce the threshold current, however its larger detuning from the maximum gain results in an overall larger threshold current. At higher injection levels, however, Device II achieves larger output power due to its higher slope efficiency. We note that the maximum of the gain is dependent on the injection current. This can be observed in the emission spectra of the solitary diode, shown in Fig. S14B. As the injection current increases, the emission undergoes a pronounced blue-shift due to the shift of the quasi-Fermi levels with carrier density (*76*). Between

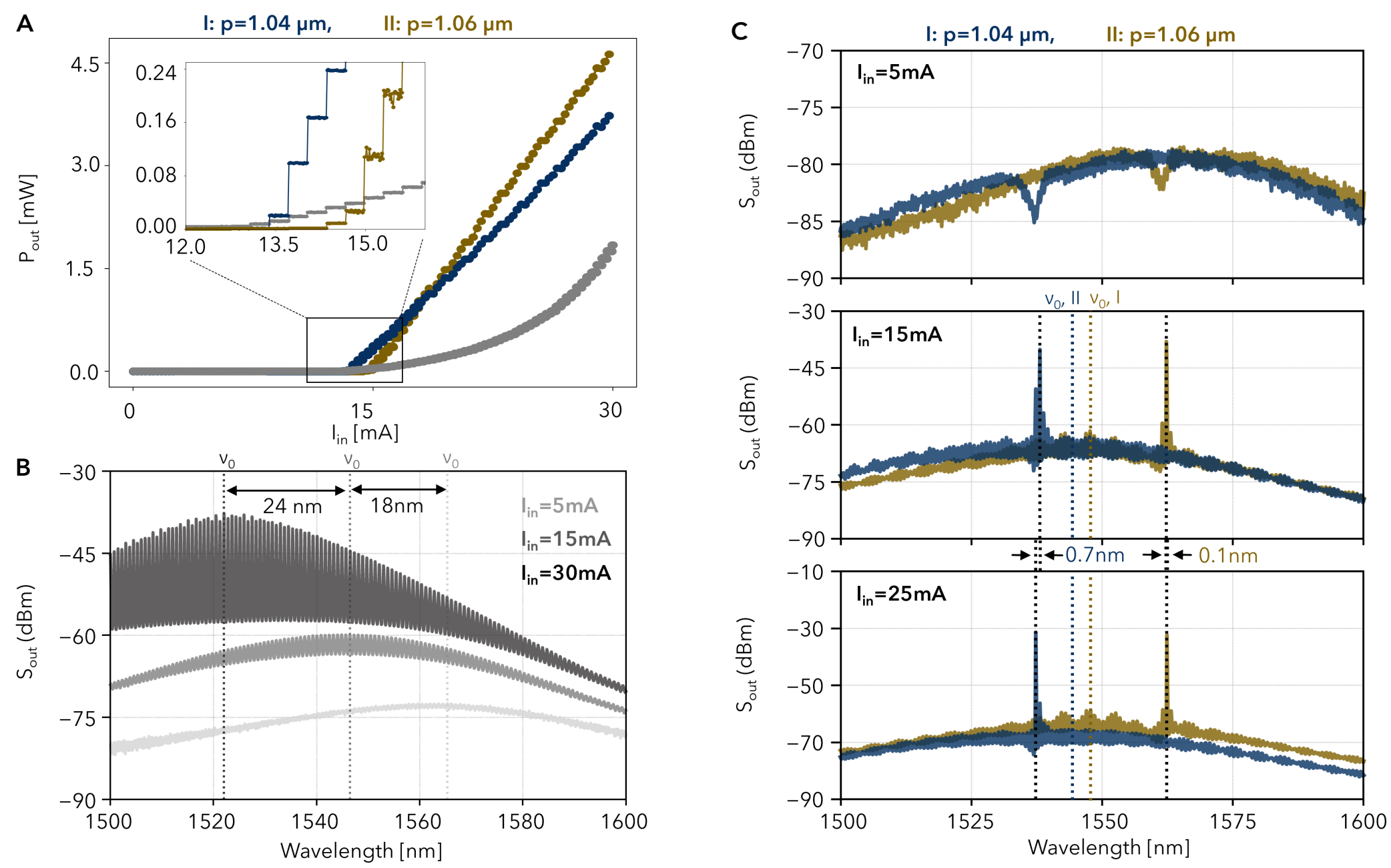

Figure S14: **Comparison of IV and spectral characteristics for external-cavity devices with different resonance wavelengths**. **A**. Output power P_{out} versus injection current I_{in} for Device I ($p = 1.04\ \mu$m, blue) and Device II ($p = 1.06\ \mu$m, gold). Device II, with higher $r_{\max}$ but resonance further from ν_0, shows a reduced threshold current. **B**. Emission spectra of the solitary diode at $I_{\text{in}} = 5$, 15, and 30 mA. The spectra exhibit a pronounced blue-shift with current, attributed to the carrier-induced shift of the quasi-Fermi levels. The total shift amounts to $\sim$ 42 nm between 5 and 30 mA. **C**. Emission spectra of the external-cavity devices under feedback for $I_{\text{in}} = 5$, 15, and 25 mA. In both devices, the lasing frequency is locked to the external-cavity resonance, and the blue-shift is strongly suppressed. The residual shift is limited to $\sim$ 0.7 nm for Device I and $\sim$ 0.1 nm for Device II, highlighting the stabilization effect of the external cavity.

$I_{\text{in}} = 5$ mA and $I_{\text{in}} = 30$ mA, the total spectral shift reaches approximately 42 nm. Overall, these results highlight the intrinsic trade-off between lasing threshold and output power: a higher $r_{\max}$ favors lower threshold, while larger detuning increases the threshold. This experimental observation is in line with our modeling (section Note 4.2 and 4.1).

In contrast to the solitary diode, the lasing frequency is pinned to the metasurface resonance when operated in the external-cavity configuration. This becomes evident when comparing the spectral shape of the two configurations (Fig. S14c). Furthermore, this frequency locking is robust across injection currents. Our metasurface design featuring one single resonance across the entire gain bandwidth serves to additionally prevent mode hopping. For Device I, the lasing wavelength shifts by only $\sim$ 0.7 nm between $I_{\text{in}} = 15$ and 25 mA, while for Device II the residual shift is further reduced to $\sim$ 0.1 nm over the same current range. These results demonstrate that the external cavity not only selects the lasing frequency, but also stabilizes it against carrier-induced detuning.

5.2 Side band analysis

An important figure of merit of any single mode laser is the side mode suppression ratio (SMSR). This ratio describes the amount by which other modes are suppressed compared to the lasing mode. In this section, we analyze the SMSR of our external cavity laser and demonstrate its robustness across injection current. We distinguish two types of SMSR, related to the internal ($\text{SMSR}_i = \min(\text{SMSR}_i^+, \text{SMSR}_i^-)$) and external cavity modes($\text{SMSR}_e = \min(\text{SMSR}_e^+, \text{SMSR}_e^-)$), respectively. These two sets of modes have different free spectral ranges of $\text{FSR}_\text{i} = 90$ GHz and $\text{FSR}_\text{e} = 5$ GHz.

Fig. S15A-B compare the measured spectra at different injection currents, including zoom-ins to visualize the external-cavity modes (highlighted in gray). For the sake of comparison, one dataset below threshold at $I_\text{in} = 12.6$ mA is also included. The current dependence of SMSR_i is shown in Fig. S15B. The evolution of SMSR_i and SMSR_e as a function of injection current is then visualized in Fig. S15D. Starting from threshold at $I_\text{in} \approx 13.6$ mA, SMSR_i decreases rapidly and reaches values as low as −55 dB at $I_\text{in} = 25$ mA, reflecting the effective suppression of internal side modes with increasing current. The external SMSR ratio SMSR_e, on the other hand, also improves with injection current, reaching better than 30 dB at $I_\text{in} = 25$ mA. This disparity between the external and internal side mode suppression ratio is related to the free spectral range of these modes. We expect that choosing shorter external cavity lengths would result in improved internal side mode suppression ratio, highlighting possible avenues to improve the performance of our platform.

A second important figure of merit is the amount of modulation provided by the metasurface to the external cavity. As shown in the main manuscript, when the metasurface modulates the intracavity field, either the instantaneous frequency or the output power are modulated. In the frequency domain, this manifests as sideband creation around the optical modes of the cavity. To investigate the robustness of this modulation efficiency across currents, we apply a modulation frequency of $\nu_{mod} = 50$ MHz and record the sideband ratio SBR which is the ratio between the RF-generated sideband and the lasing peak (Fig. S15C). When tracking it as a function of injection current (Fig. S15D) we notice a stable response up to 21 mA. At this current, mode hopping occurs between two adjacent internal cavity modes (see Fig. S15A). After the hop, SBR partially recovers at higher currents. This effect could be mitigated by either having better anti-reflection coatings or

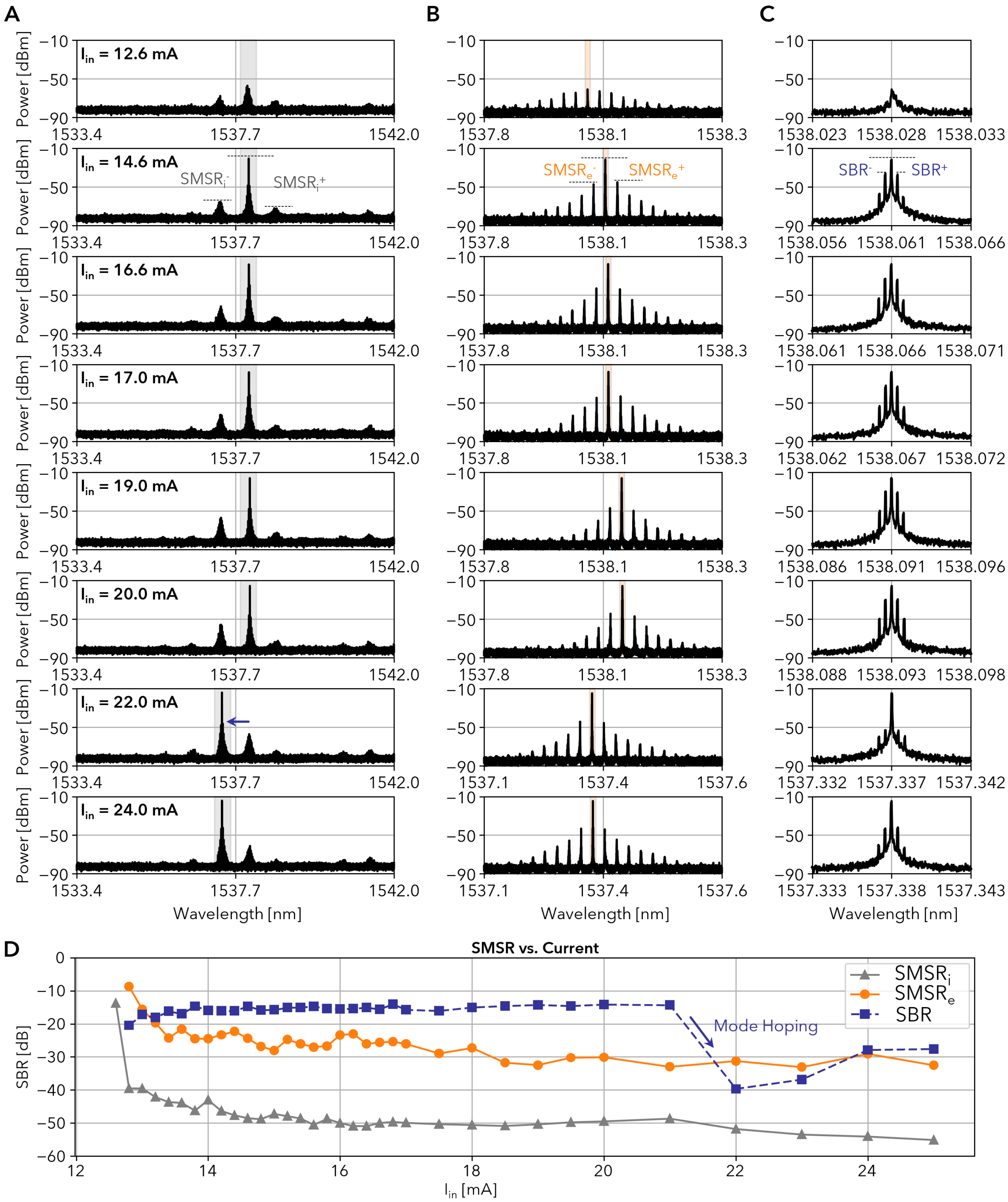

Figure S15: **Side band ratio analysis**. **A-C**. Optical spectra recorded at different injection currents, including a dataset below threshold at $I_{\text{in}} = 12.6$ mA for comparison. Grey shading highlights the external-cavity modes, while the orange zoom-in marks the main peak of the external mode and the RF sidebands around it at $\nu_m = 50$ MHz. **D**. Current dependence of the internal sidemode suppression ratio SMSR$_i$, the external sidemode suppression ratio SMSR$_e$, and the modulation sideband ratio SBR. SMSR$_i$ decreases steadily with current, reaching −55 dB at $I_{\text{in}} = 25$ mA, indicating strong suppression of internal sidebands. SMSR$_e$ remains relatively stable around 30−35 dB, confirming robust suppression of neighboring cavity modes. SBR stays above 20 dB across most of the operating range but drops abruptly at $I_{\text{in}} \approx 22$ mA. The zoomed-in spectra in (A) show that this discontinuity coincides with a mode hop, visible as a sudden change in lasing frequency and sideband strength. After the hop, SBR partially recovers at higher currents.

opting for metasurfaces with higher r_{max}, which may however compromise output power.

5.3 Frequency tuning

In this section, we provide further data related to the frequency tuning using our platform. The instantaneous frequency used to characterize the frequency modulation is obtained from the heterodyne beat signal using a short-time Fourier transform (STFT). The STFT analysis is subject to the fundamental time–frequency uncertainty relation, which imposes a trade-off between temporal and spectral resolution. In particular, the achievable frequency resolution is approximately given by $\Delta f \sim \frac{1}{\Delta t}$ where Δt is the temporal window used in the STFT analysis. In our experiments, the peak-to-peak frequency excursion reaches approximately 100 MHz for a 10 V drive. For a modulation frequency of 1 MHz (corresponding to a modulation period of 1 μs), we used a temporal analysis window of 100 ns, which provides approximately ten sampling points per modulation period. This choice allows reliable reconstruction of the instantaneous frequency trajectory while maintaining sufficient spectral resolution. Increasing the modulation frequency would require proportionally shorter analysis windows in order to resolve the temporal evolution of the chirp. However, reducing the window length would simultaneously degrade the frequency resolution of the STFT, resulting in spectral broadening and inaccurate extraction of the instantaneous frequency. For this reason, reliable reconstruction of the chirp waveform using the present analysis approach becomes increasingly challenging beyond modulation frequencies of approximately 1 MHz. Importantly, this limitation arises from the signal processing method used to extract the instantaneous frequency and does not reflect the intrinsic bandwidth of the electro-optic metasurface.

While Fig. 3 in the main manuscript showed electro-optic frequency tuning for an injection current of $I_{\text{in}} = 16$ mA, here we also investigate the corresponding frequency deviation from an ideal triangular waveform in detail, shown in Fig. S16. For each modulation frequency, Fig. S16A shows the heterodyne spectrogram of the beat signal over multiple modulation periods, and Fig. S16B plots the corresponding frequency deviation from an ideal triangular waveform. We find that slow-frequency drifts impact the linearity stronger at low modulation frequencies. At higher modulation frequencies (above 250 kHz), the RMS deviations remain below 10 MHz at frequencies. The RMS deviation reported in Fig. 3D quantifies the deviation of the measured

instantaneous frequency from an ideal triangular waveform under electro-optic modulation.

The instantaneous frequency $\nu_{\text{inst}}(t)$ is extracted by tracking the maximum intensity of the spectrogram of the heterodyne beat signal as a function of time. An ideal triangular waveform $\nu_{\text{tri}}(t)$ is constructed with the same time sampling as the experimental data and with the same peak-to-peak frequency excursion, independently for each modulation frequency.

The RMS deviation is then defined as

$$\text{RMS} = \sqrt{\frac{1}{N}\sum_{i=1}^{N}\left[\nu_{\text{inst}}(t_i) - \nu_{\text{tri}}(t_i)\right]^2}. \tag{S9}$$

This metric quantifies the overall fidelity of the frequency chirp produced by the device. The RMS deviation therefore captures multiple contributions. First, it includes fluctuations of the instantaneous laser frequency (i.e. frequency noise of the cavity), since these are uncorrelated with the applied triangular drive and lead to deviations from the ideal waveform. Second, it can also reflect non-idealities in the electro-optic response itself, for example if the frequency response to the applied voltage deviates from perfect linearity due to RF bandwidth limitations or intrinsic device dynamics. In our measurements, the frequency-noise analysis shows that the laser noise decreases with offset frequency, reaching an instantaneous linewidth of approximately 1.8 MHz at 100 kHz offset frequency (See Fig. S17C). This behavior is also reflected in the RMS curve shown in Fig. 3d of the main text, which reaches a minimum value of approximately 3 MHz at modulation frequencies around 500 kHz. At lower modulation frequencies, slow mechanical fluctuations of the external cavity length introduce additional deviations from the ideal triangular waveform, leading to an increase of the RMS value.

5.4 Instantaneous linewidth extraction

The instantaneous linewidth is derived from the high-frequency white frequency-noise plateau of the single-sided frequency-noise power spectral density $S_\nu(f)$. To retrieve this quantity, we perform heterodyne beat measurements where we use a stable laser as a local oscillator. Beating our metasurface-enabled external cavity laser with a DFB on a fast photodiode (PD100 Low noise photodetector) yields a beat signal that we record as a function of time with a digital-analog

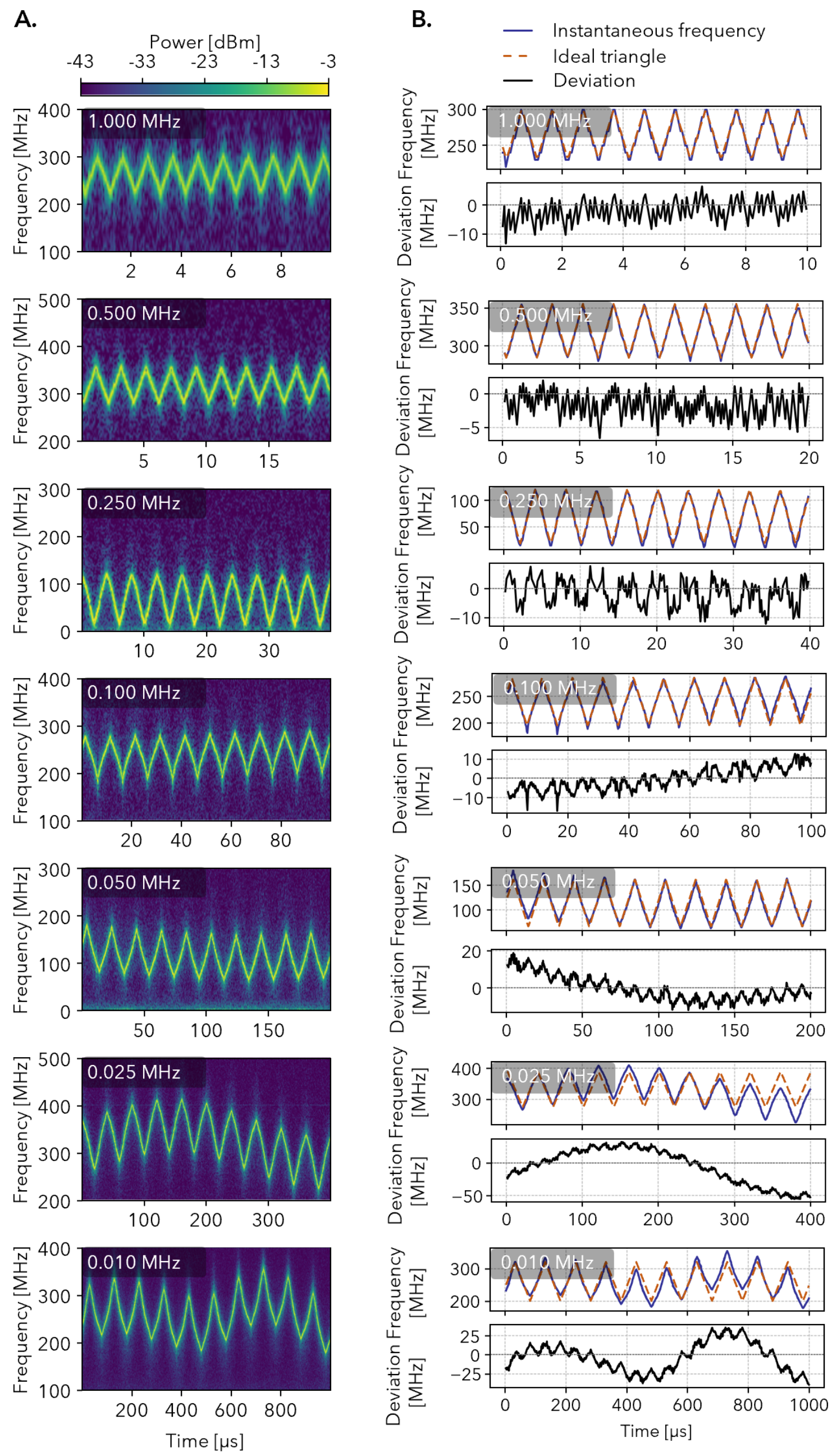

Figure S16: **Extended electro-optic frequency tuning measurements**. **A**. Spectrograms of the heterodyne beat signal recorded at I_{in} = 16 mA for modulation frequencies ν_{mod} ranging from 10 kHz to 1 MHz. **B**. Corresponding instantaneous frequency traces (top panels) together with the ideal triangular waveform (dashed) and the deviation from the ideal triangle (bottom panels) for each modulation frequency. These results extend the main-text measurements by providing the full spectrograms and deviation analysis across a broad range of modulation frequencies.

acquisition card with sufficient bandwidth (Spectrum Instrumentation, M4i.2442-x8 digitizer, 250 MHz). From the time-resolved heterodyne signal, we extract the instantaneous beat frequency

$\nu_{beat}(t)$ by tracking the ridge of the time–frequency representation. The frequency fluctuations are defined as

$$\delta\nu(t) = \nu_{beat}(t) - \langle \nu_{beat}(t) \rangle. \tag{S10}$$

The single-sided frequency-noise power spectral density (PSD), $S_\nu(f)$, is obtained from the Fourier transform of $\delta\nu(t)$:

$$S_\nu(f) = |\mathcal{F}\{\delta\nu(t)\}|^2. \tag{S11}$$

In the high-frequency region, the frequency-noise spectrum reaches a flat plateau corresponding to white frequency noise. In this region,

$$S_\nu(f) \approx S_{\nu,0}, \tag{S12}$$

where $S_{\nu,0}$ is frequency-independent. White frequency noise leads to a Lorentzian optical lineshape with full width at half maximum (FWHM)

$$\Delta\nu_{\text{inst}} = \pi S_{\nu,0}. \tag{S13}$$

Experimentally, $S_{\nu,0}$ is determined by taking the median value of $S_\nu(f)$ within the flat plateau region. Our results of this procedure are shown in Fig. S17. We use a DFB laser (Youopto, YTFCDD02BK1IH-1550) as a local oscillator, which is the most stable laser available to us with a linewidth of 100 kHz. To confirm that the stability of the DFB is sufficient to measure the linewidth of our external cavity laser, we perform a control experiment where we beat two identical DFBs with slightly offset frequencies (see experimental setup in Fig. S17A(a) and results in Fig. S17B(a)). We then perform a second experiment where one DFB is replaced by our metasurface-enabled external cavity laser (see experimental setup in Fig. S17A(b) and results in Fig. S17B(b)). We track both beats over the time span of 1 s, and notice that the beat of the two DFBs is markedly quieter than the beat between our metasurface-controlled external cavity and a single DFB, confirming that the stability of the local oscillator is not limiting our analysis. We then extract the instantaneous beat frequency $\nu_{beat}(t)$ by tracking the maximum of the the time-frequency spectrogram as a function of time and compute the corresponding single-sided frequency-noise power spectral density $S_\nu(f)$ for both cases (see Fig. S17C).

By analysing the single-sided frequency-noise power spectral density $S_\nu(f)$ in Fig. S17C, we notice that, unlike the DFB, which is a monolithic laser with no mechanical parts, our external

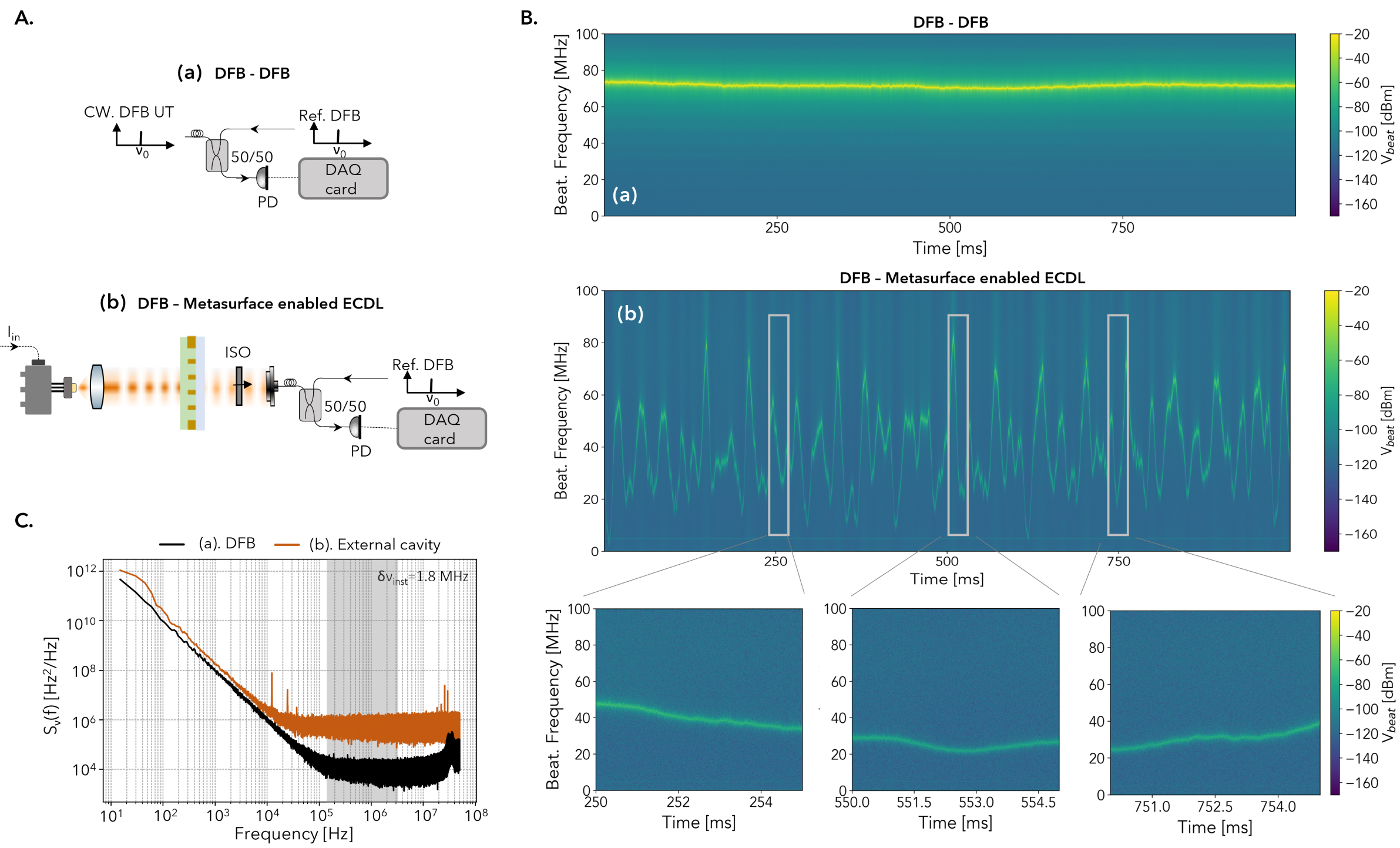

Figure S17: **Instantaneous linewidth characterization via heterodyne beat measurements.** **A**. Experimental configuration. (a) Reference measurement that beats two similar DFB laser lines to determine the DFB linewidth. (b) Heterodyne measurement that beats the external cavity laser line and a stable DFB reference laser. The beat signal is detected using a fast photodiode and recorded with a data acquisition system. **B**. Panels B(a) and B(b) report the spectrograms calculated from the heterodyne beat signals acquired in case A(a) and A(b). In case B(a) the trace reveals a stable and narrow frequency trace that is the intrinsic characteristic of the reference DFB lasers, whereas case (b) features larger frequency fluctuations exhibited by our external cavity. **C**. Frequency-noise power spectral density $S_\nu(f)$ extracted from the ridge of the beat spectrogram for the external cavity laser (orange) and the DFB reference (black). The high-frequency white-noise plateau (shaded region) is used to determine the instantaneous linewidth according to $\Delta\nu_{\text{inst}} = \pi S_{\nu,0}$. From the white frequency-noise level, we obtain an instantaneous linewidth of approximately 1.8 MHz for the external cavity laser.

cavity has more pronounced noise at a few 10s of Hz to a few 10s of kHz offset frequency (also visible in the few millisecond-long zoom-ins of Fig. S17B(b)). We attribute this to mechanical vibrations of the optomechanical pieces (posts and holders) used in our external cavity. To confirm this hypothesis, we test our external cavity also against the high-end external cavity tunable laser (N9997C) from Keysight which we also characterize on millisecond time-scales, and the results are shown in Fig. S18. The commercial laser also has strong variations of the instantaneous frequency

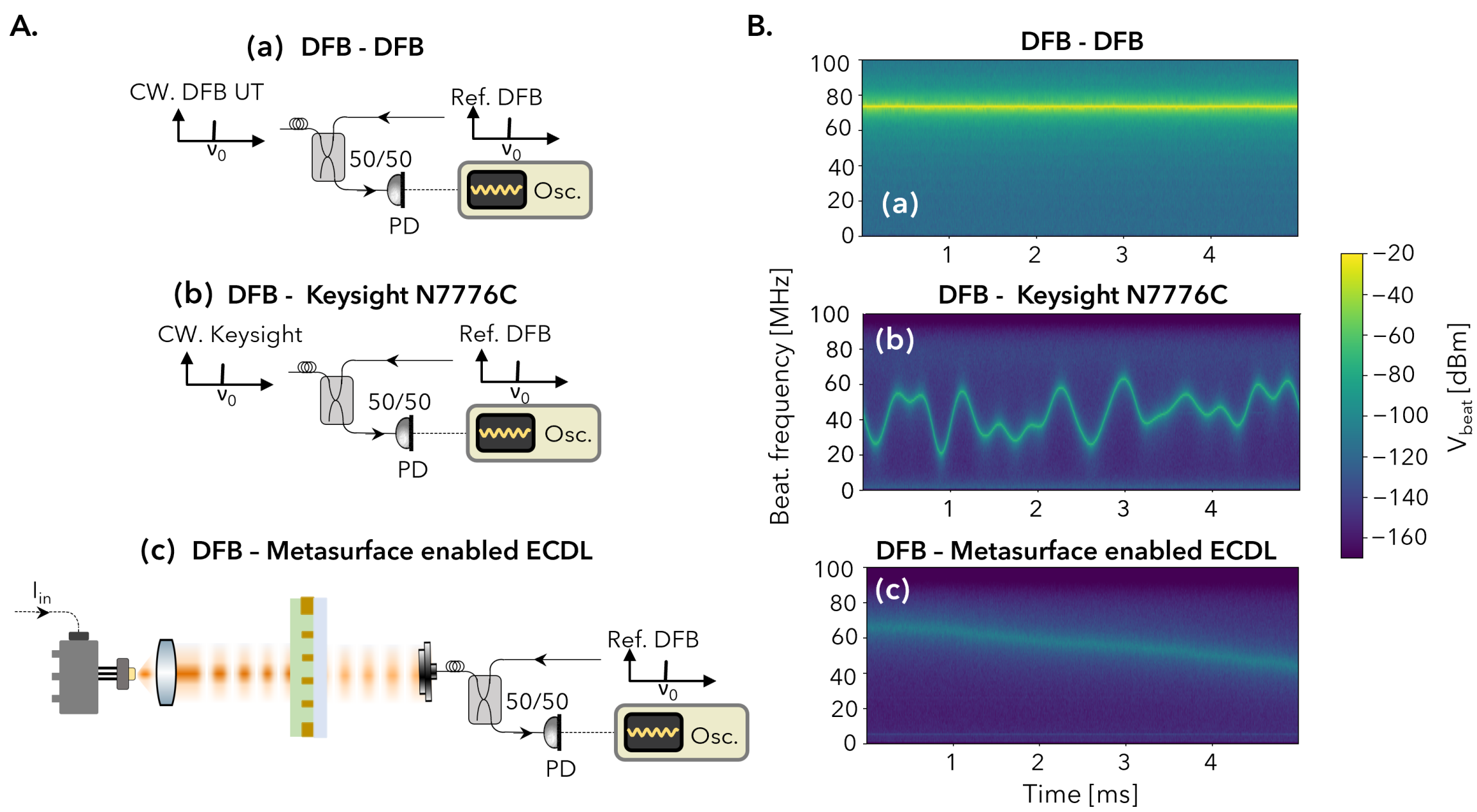

Figure S18: **Comparison of slow beatnote fluctuations in different external cavity configurations.** **A**. Heterodyne measurement setups: (a) reference measurement between two DFB lasers; (b) commercial Keysight tunable external cavity laser; (c) metasurface-based external cavity laser presented in this work. In all cases, the device under test is heterodyned with a stable DFB reference laser and the beat signal is recorded with an oscilloscope. **B**. Time–frequency spectrograms of the corresponding beat notes. While all free-space external cavity systems exhibit slow offset fluctuations due to environmental cavity-length perturbations, the metasurface-based external cavity (c) shows comparable or lower fluctuation frequencies. These slow drifts primarily affect the beat-note offset.

reaching several 10s of MHz over a few milliseconds, similar to our implementation (Fig. S18).

In the high-frequency region, the single-sided frequency-noise power spectral density $S_\nu(f)$ of our metasurface-enabled external cavity laser reaches a white frequency-noise plateau $S_{\nu,0}$ (in this case around 100 kHz, highlighted by the gray window in Fig. S17C). We also note that the single-sided frequency-noise power spectral density $S_\nu(f)$ of the two DFBs is about one order of magnitude lower than the one of the DFB and the metasurface external cavity laser. Consequently, the contribution of the DFB to the white frequency-noise plateau $S_{\nu,0}$ of can be neglected for the DFB-external cavity beat, and the magnitude of this plateau can be used to extract the instantaneous linewidth (FWHM) of our laser as follows

$$\Delta\nu_{\text{inst}} = \pi S_{\nu,0}. \tag{S14}$$

Using this procedure, we obtain an instantaneous linewidth of approximately

$$\Delta\nu_{\text{inst}} \approx 1.8 \times 10^6 \text{ Hz}.$$

5.5 Beam profile characterization

The incident polarization is oriented perpendicular to the subwavelength gold electrodes (200 nm wide), minimizing direct coupling to plasmonic or scattering modes associated with the metallic structures. As a result, the electrodes primarily enable the guided-mode resonance with minimal additional loss. To confirm the impact on the beam quality, we utilize an InGaAs camera and image the beam profile after the laser in three different configurations: (I) solitary diode operation, (II) feedback from the metasurface, and (III) feedback from the substrate coated by JRD1. Fig. S19 shows the beam images for the three configurations investigated, together with a schematic of the measurement setup. We observe a similar beam with a round shape in all three configurations, while diffraction rings are visible in all cases, suggesting that the beam is slightly clipped by the lens focusing onto the metasurface. The diffraction rings appear more pronounced in the configurations with optical feedback. However, this difference primarily arises from variations in the camera calibration, in particular the integration time, which was adjusted to account for different output power levels in each configuration. All images were acquired above threshold, but not under identical detection conditions, and therefore the relative visibility of the rings should not be interpreted as a change in the intrinsic beam quality. To further quantify the beam quality, we performed knife-edge measurements at the focal plane. For the configuration with feedback from the metasurface (case II), we extract a beam quality factor of $M^2 = 2.06$, indicating a deviation from an ideal Gaussian beam. Unfortunately, a larger lens could not be accommodated within our compact setup, but we envision mitigating these space/footprint constraints using flat lenses in combination with our electro-optic metasurface in the future (*22, 100*).

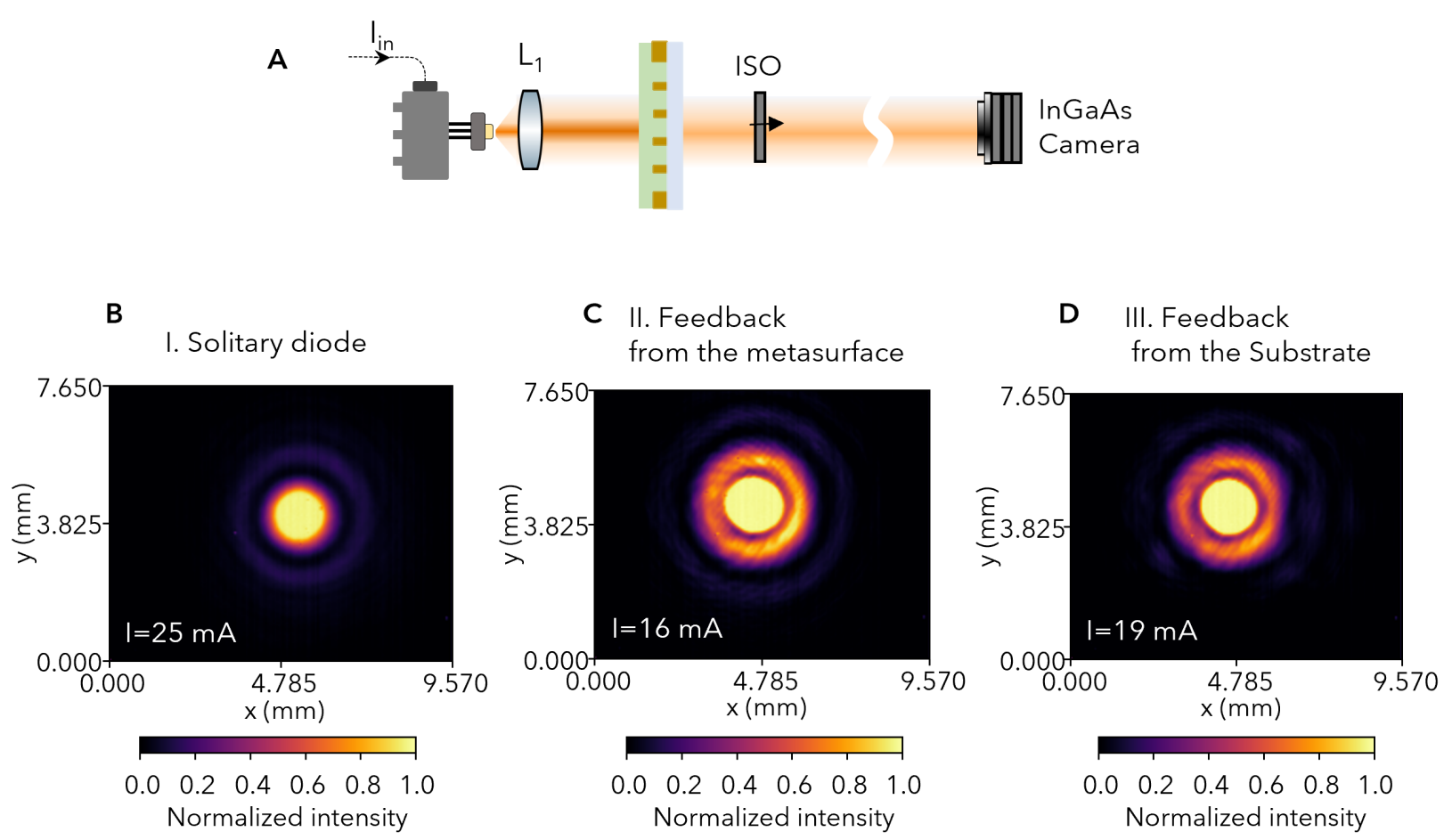

Figure S19: **Beam profiles for different operating configurations of the external-cavity system. A**. Schematic of the experimental setup used to image the output beam with an InGaAs camera. **B**. Solitary diode operation. **C**. Optical feedback from the metasurface. **D**. Optical feedback from the substrate coated by the JRD1 layer. The images show that the beam remains circular and well-defined in all configurations. Diffraction rings are observed in all cases and are attributed to partial clipping of the beam by the focusing optics rather than to the metasurface itself. We note that the images are purposely saturated to clearly show the diffraction rings around it.

REFERENCES

1. D. Liang, C. Zhang, P. Zhang, S. Liu, H. Li, S. Niu, R. Z. Rao, L. Zhao, X. Chen, H. Li, Y. Huo, Evolution of laser technology for automotive LiDAR, an industrial viewpoint. *Nat. Commun.* **15**, 7660 (2024).

2. X. Zhang, K. Kwon, J. Henriksson, J. Luo, M. C. Wu, A large-scale microelectromechanical-systems-based silicon photonics LiDAR. *Nature* **603**, 253–258 (2022).

3. F. R. Giorgetta, I. Coddington, E. Baumann, W. C. Swann, N. R. Newbury, Fast high-resolution spectroscopy of dynamic continuous-wave laser sources. *Nat. Photonics* **4**, 853–857 (2010).

4. V. S. Letokhov, Nonlinear high resolution laser spectroscopy. *Science* **190**, 344–351 (1975).

5. A. E. Willner, K. Pang, H. Song, K. Zou, H. Zhou, Orbital angular momentum of light for communications. *Appl. Phys. Rev.* **8**, 041312 (2021).

6. A. Liu, P. Wolf, J. A. Lott, D. Bimberg, Vertical-cavity surface-emitting lasers for data communication and sensing. *Photonics Res.* **7**, 121–136 (2019).

7. J. Wang, J.-Y. Yang, I. M. Fazal, N. Ahmed, Y. Yan, H. Huang, Y. Ren, Y. Yue, S. Dolinar, M. Tur, A. E. Willner, Terabit free-space data transmission employing orbital angular momentum multiplexing. *Nat. Photonics* **6**, 488–496 (2012).

8. L. Jonušauskas, D. Gailevičius, S. Rekštytė, T. Baldacchini, S. Juodkazis, M. Malinauskas, Mesoscale laser 3D printing. *Opt. Express* **27**, 15205–15221 (2019).

9. C. Voumard, External-cavity-controlled 32-MHz narrow-band cw GaAlAs-diode lasers. *Opt. Lett.* **1**, 61–63 (1977).

10. K. C. Harvey, C. J. Myatt, External-cavity diode laser using a grazing-incidence diffraction grating. *Opt. Lett.* **16**, 910–912 (1991).

11. R. Lang, K. Kobayashi, External optical feedback effects on semiconductor injection laser properties. *IEEE J. Quantum Electron.* **16**, 347–355 (1980).

12. D. A. S. Heim, D. Bose, K. Liu, A. Isichenko, D. J. Blumenthal, Hybrid integrated ultra-low linewidth coil stabilized isolator-free widely tunable external cavity laser. *Nat. Commun.* **16**, 5944 (2025).

13. G. Lihachev, J. Riemensberger, W. Weng, J. Liu, H. Tian, A. Siddharth, V. Snigirev, V. Shadymov, A. Voloshin, R. N. Wang, J. He, S. A. Bhave, T. J. Kippenberg, Low-noise frequency-agile photonic integrated lasers for coherent ranging. *Nat. Commun.* **13** , 3522 (2022).

14. M. Li, L. Chang, L. Wu, J. Staffa, J. Ling, U. A. Javid, S. Xue, Y. He, R. Lopez-Rios, T. J. Morin, H. Wang, B. Shen, S. Zeng, L. Zhu, K. J. Vahala, J. E. Bowers, Q. Lin, Integrated Pockels laser. *Nat. Commun.* **13**, 5344 (2022).

15. B. Wacogne, J.-P. Goedgebuer, H. Porte, Single lithium niobate crystal for mode selection and phase modulation in a tunable extended-cavity laser diode. *Opt. Lett.* **19**, 1334–1336 (1994).

16. K. S. Repasky, G. W. Switzer, J. L. Carlsten, Design and performance of a frequency chirped external cavity diode laser. *Rev. Sci. Instrum.* **73**, 3154–3159 (2002).

17. A. Schremer, C. L. Tang, Single-frequency tunable external-cavity semiconductor laser using an electro-optic birefringent modulator. *Appl. Phys. Lett.* **55**, 19–21 (1989).

18. C. Ye, *Tunable External Cavity Diode Lasers* (World Scientific, Singapore & Hackensack, NJ, 2007).

19. W. Liang, V. S. Ilchenko, D. Eliyahu, A. A. Savchenkov, A. B. Matsko, D. Seidel, L. Maleki, Ultralow noise miniature external cavity semiconductor laser. *Nat. Commun.* **6**, 7371 (2015).

20. M. Ossiander, M. L. Meretska, S. Rourke, C. Spägele, X. Yin, I.-C. Benea-Chelmus, F. Capasso, Metasurface-stabilized optical microcavities. *Nat. Commun.* **14**, 1114 (2023).

21. J.-L. Wu, Y.-Q. Xu, J.-J. Xu, X.-M. Wei, A. Chan, A. H. Tang, A. K. Lau, B. M. Chung, H. Cheung Shum, E. Y. Lam, K. K. Wong, K. K. Tsia, Ultrafast laser-scanning time-stretch imaging at visible wavelengths. *Light Sci. Appl.* **6**, e16196 (2017).

22. Y. Kim, T. Choi, G.-Y. Lee, C. Kim, J. Bang, J. Jang, Y. Jeong, B. Lee, Metasurface folded lens system for ultrathin cameras. *Sci. Adv.* **10**, eadr2319 (2024).

23. O. Reshef, M. P. DelMastro, K. K. M. Bearne, A. H. Alhulaymi, L. Giner, R. W. Boyd, J. S. Lundeen, An optic to replace space and its application towards ultra-thin imaging systems. *Nat. Commun.* **12**, 3512 (2021).

24. K. Zou, K. Pang, H. Song, J. Fan, Z. Zhao, H. Song, R. Zhang, H. Zhou, A. Minoofar, C. Liu, X. Su, A. McClung, M. Torfeh, A. Arbabi, M. Tur, A. E. Willner, High-capacity free-space optical communications using wavelength-and mode-division-multiplexing in the mid-infrared region. *Nat. Commun.* **13**, 7662 (2022).

25. I. Kim, R. J. Martins, J. Jang, T. Badloe, S. Khadir, H.-Y. Jung, H. Kim, J. Kim, P. Genevet, J. Rho, Nanophotonics for light detection and ranging technology. *Nat. Nanotechnol.* **16**, 508–524 (2021).

26. J. Park, B. G. Jeong, S. I. Kim, D. Lee, J. Kim, C. Shin, C. B. Lee, T. Otsuka, J. Kyoung, S. Kim, K.-Y. Yang, Y.-Y. Park, J. Lee, I. Hwang, J. Jang, S. H. Song, M. L. Brongersma, K. Ha, S.-W. Hwang, H. Choo, B. L. Choi, All-solid-state spatial light modulator with independent phase and amplitude control for three-dimensional LiDAR applications. *Nat. Nanotechnol.* **16**, 69–76 (2021).

27. Z. Dong, Y. Ling, Y. Li, Y. Su, Motion hologram: Jointly optimized hologram generation and motion planning for photorealistic 3D displays via reinforcement learning. *Sci. Adv.* **11**, eads9876 (2025).

28. J. Yang, L. S. Li, Q. He, C. Li, Y. Qu, L. V. Wang, An ultrahigh-fidelity 3D holographic display using scattering to homogenize the angular spectrum. *Sci. Adv.* **9**, eadi9987 (2023).

29. J. Bütow, J. S. Eismann, V. Sharma, D. Brandmüller, P. Banzer, Generating free-space structured light with programmable integrated photonics. *Nat. Photonics* **18**, 243–249 (2024).

30. J. Yang, C. Zou, C. Lin, Y. Zhu, Y. Zhu, F. Dang, Y. Yuan, Z. Yu, Y. Wang, Y. Qin, Noise compensation methods for optical fiber frequency sweeping interferometry: A review. *J. Lightwave Technol.* **41**, 4035–4050 (2022).

31. G. Rizzelli, A. Nespola, S. Straullu, F. Forghieri, R. Gaudino, Phase noise impact and scalability of self-homodyne short-reach coherent transmission using DFB lasers. *J. Lightwave Technol.* **40**, 37–44 (2022).

32. A. V. T. Cartaxo, B. Wedding, W. Idler, Influence of fiber nonlinearity on the phase noise to intensity noise conversion in fiber transmission: Theoretical and experimental analysis. *J. Lightwave Technol.* **16**, 1187–1194 (2002).

33. A. M. Shaltout, K. G. Lagoudakis, J. van de Groep, S. J. Kim, J. Vučković, V. M. Shalaev, M. L. Brongersma, Spatiotemporal light control with frequency-gradient metasurfaces. *Science* **365**, 374–377 (2019).

34. A. Arbabi, E. Arbabi, Y. Horie, S. M. Kamali, A. Faraon, Planar metasurface retroreflector. *Nat. Photonics* **11**, 415–420 (2017).

35. F. Tang, J. Wu, T. Albrow-Owen, H. Cui, F. Chen, Y. Shi, L. Zou, J. Chen, X. Guo, Y. Sun, J. Luo, B. Ju, J. Huang, S. Liu, B. Li, L. Yang, E. A. Munro, W. Zheng, H. J. Joyce, H. Chen, L. Che, S. Dong, Z. Sun, T. Hasan, X. Ye, Y. Yang, Y. Zongyin, Metasurface spectrometers beyond resolution-sensitivity constraints. *Sci. Adv.* **10**, eadr7155 (2024).

36. W. T. Chen, A. Y. Zhu, F. Capasso, Flat optics with dispersion-engineered metasurfaces. *Nat. Rev. Mater.* **5**, 604–620 (2020).

37. N. A. Rubin, G. D'Aversa, P. Chevalier, Z. Shi, W. T. Chen, F. Capasso, Matrix Fourier optics enables a compact full-Stokes polarization camera. *Science* **365**, eaax1839 (2019).

38. F. Aieta, M. A. Kats, P. Genevet, F. Capasso, Multiwavelength achromatic metasurfaces by dispersive phase compensation. *Science* **347**, 1342–1345 (2015).

39. N. Yu, F. Aieta, P. Genevet, M. A. Kats, Z. Gaburro, F. Capasso, A broadband, background-free quarter-wave plate based on plasmonic metasurfaces. *Nano Lett.* **12**, 6328–6333 (2012).

40. A. V. Kildishev, A. Boltasseva, V. M. Shalaev, Planar photonics with metasurfaces. *Science* **339**, 1232009 (2013).

41. W. Meng, J. E. Fröch, K. Cheng, D. Pi, B. Li, A. Majumdar, S. A. Maier, H. Ren, M. Gu, X. Fang, Ultranarrow-linewidth wavelength-vortex metasurface holography. *Sci. Adv.* **11**, eadt9159 (2025).

42. J. Li, S. Kamin, G. Zheng, F. Neubrech, S. Zhang, N. Liu, Addressable metasurfaces for dynamic holography and optical information encryption. *Sci. Adv.* **4**, eaar6768 (2018).

43. C. Meng, P. C. V. Thrane, F. Ding, J. Gjessing, M. Thomaschewski, C. Wu, C. Dirdal, S. I. Bozhevolnyi, Dynamic piezoelectric MEMS-based optical metasurfaces. *Sci. Adv.* **7**, eabg5639 (2021).

44. A. Forbes, Structured light from lasers. *Laser Photonics Rev.* **13**, 1900140 (2019).

45. C. Huang, C. Zhang, S. Xiao, Y. Wang, Y. Fan, Y. Liu, N. Zhang, G. Qu, H. Ji, J. Han, Li Ge, Y. Kivshar, Q. Song, Ultrafast control of vortex microlasers. *Science* **367**, 1018–1021 (2020).

46. H. Zhong, Y. Yu, Z. Zheng, Z. Ding, X. Zhao, J. Yang, Y. Wei, Y. Chen, S. Yu, Ultra-low threshold continuous-wave quantum dot mini-BIC lasers. *Light Sci. Appl.* **12**, 100 (2023).

47. M.-S. Hwang, H.-C. Lee, K.-H. Kim, K.-Y. Jeong, S.-H. Kwon, K. Koshelev, Y. Kivshar, H.-G. Park, Ultralow-threshold laser using super-bound states in the continuum. *Nat. Commun.* **12**, 4135 (2021).

48. L. Xu, D. Chen, C. A. Curwen, M. Memarian, J. L. Reno, T. Itoh, B. S. Williams, Metasurface quantum-cascade laser with electrically switchable polarization. *Optica* **4**, 468–475 (2017).

49. C. A. Curwen, J. L. Reno, B. S. Williams, Broadband continuous single-mode tuning of a short-cavity quantum-cascade VECSEL. *Nat. Photonics* **13**, 855–859 (2019).

50. L. Xu, C. A. Curwen, D. Chen, J. L. Reno, T. Itoh, B. S. Williams, Terahertz metasurface quantum-cascade VECSELs: Theory and performance. *IEEE J. Sel. Top. Quantum Electron.* **23**, 1–12 (2017).

51. L. Xu, C. A. Curwen, P. W. C. Hon, Q.-S. Chen, T. Itoh, B. S. Williams, Metasurface external cavity laser. *Appl. Phys. Lett.* **107**, 221105 (2015).

52. P.-N. Ni, P. Fu, P.-P. Chen, C. Xu, Y.-Y. Xie, P. Genevet, Spin-decoupling of vertical cavity surface-emitting lasers with complete phase modulation using on-chip integrated Jones matrix metasurfaces. *Nat. Commun.* **13**, 7795 (2022).

53. Y.-Y. Xie, P.-N. Ni, Q.-H. Wang, Q. Kan, G. Briere, P.-P. Chen, Z.-Z. Zhao, A. Delga, H.-R. Ren, H.-D. Chen, C. Xu, P. Genevet, Metasurface-integrated vertical cavity surface-emitting lasers for programmable directional lasing emissions. *Nat. Nanotechnol.* **15**, 125–130 (2020).

54. H.-Y. Luan, Y.-H. Ouyang, Z.-W. Zhao, W.-Z. Mao, R.-M. Ma, Reconfigurable moiré nanolaser arrays with phase synchronization. *Nature* **624**, 282–288 (2023).

55. Z.-Q. Yang, Z.-K. Shao, H.-Z. Chen, X.-R. Mao, R.-M. Ma, Spin-momentum-locked edge mode for topological vortex lasing. *Phys. Rev. Lett.* **125**, 013903 (2020).

56. M. Yoshida, S. Katsuno, T. Inoue, J. Gelleta, K. Izumi, M. De Zoysa, K. Ishizaki, S. Noda, High-brightness scalable continuous-wave single-mode photonic-crystal laser. *Nature* **618**, 727–732 (2023).

57. C. Spägele, M. Tamagnone, D. Kazakov, M. Ossiander, M. Piccardo, F. Capasso, Multifunctional wide-angle optics and lasing based on supercell metasurfaces. *Nat. Commun.* **12**, 3787 (2021).

58. A. M. Shaltout, V. M. Shalaev, M. L. Brongersma, Spatiotemporal light control with active metasurfaces. *Science* **364**, eaat3100 (2019).

59. C. Jung, E. Lee, J. Rho, The rise of electrically tunable metasurfaces. *Sci. Adv.* **10**, eado8964 (2024).

60. T. Gu, H. J. Kim, C. Rivero-Baleine, J. Hu, Reconfigurable metasurfaces towards commercial success. *Nat. Photonics* **17**, 48–58 (2023).

61. P. C. Wu, R. A. Pala, G. Kafaie Shirmanesh, W.-H. Cheng, R. Sokhoyan, M. Grajower, M. Z. Alam, D. Lee, H. A. Atwater, Dynamic beam steering with all-dielectric electro-optic III–V multiple-quantum-well metasurfaces. *Nat. Commun.* **10**, 3654 (2019).

62. H. Weigand, V. V. Vogler-Neuling, M. R. Escalé, D. Pohl, F. U. Richter, A. Karvounis, F. Timpu, R. Grange, Enhanced electro-optic modulation in resonant metasurfaces of lithium niobate. *ACS Photonics* **8**, 3004–3009 (2021).

63. A. Di Francescantonio, A. Sabatti, H. Weigand, E. Bailly-Rioufreyt, M. A. Vincenti, L. Carletti, J. Kellner, A. Zilli, M. Finazzi, M. Celebrano, R. Grange, Efficient GHz electro-optical modulation with a nonlocal lithium niobate metasurface in the linear and nonlinear regime. *Nat. Commun.* **16**, 7000 (2025).

64. S. Dagli, J. Shim, H. Carr Delgado, H. B. Balch, S. Abdollahramezani, C.-Y. Chen, V. Dolia, E. Klopfer, J. Dixon, J. Hu, B. Ogunlade, J.-H. Song, M. L. Brongersma, D. Barton, J. A. Dionne, GHz-speed wavefront shaping metasurface modulators enabled by resonant electro-optic nanoantennas. *Adv. Mater.* **37**, e06790 (2025).

65. H. Xu, F. Liu, D. L. Elder, L. E. Johnson, Y. de Coene, K. Clays, B. H. Robinson, L. R. Dalton, Ultrahigh electro-optic coefficients, high index of refraction, and long-term stability from Diels–Alder cross-linkable binary molecular glasses. *Chem. Mater.* **32**, 1408–1421 (2020).

66. I.-C. Benea-Chelmus, M. L. Meretska, D. L. Elder, M. Tamagnone, L. R. Dalton, F. Capasso, Electro-optic spatial light modulator from an engineered organic layer. *Nat. Commun.* **12**, 5928 (2021).

67. I.-C. Benea-Chelmus, S. Mason, M. L. Meretska, D. L. Elder, D. Kazakov, A. Shams-Ansari, L. R. Dalton, F. Capasso, Gigahertz free-space electro-optic modulators based on Mie resonances. *Nat. Commun.* **13**, 3170 (2022).

68. A. Yariv, *Quantum Electronics* (Wiley, ed. 3, 1989), chap. 14.6.

69. S. S. Wang, R. Magnusson, Theory and applications of guided-mode resonance filters. *Appl. Opt.* **32**, 2606–2613 (1993).

70. P. W. Milonni, J. H. Eberly, *Laser Physics* (Wiley, 2010), chap. 3–6.

71. G. R. Gray, R. Roy, Bistability and mode hopping in a semiconductor laser. *J. Opt. Soc. Am. B* **8**, 632–638 (1991).

72. N. Ogasawara, R. Ito, Output power change associated with longitudinal mode jumping in semiconductor injection lasers. *Jpn. J. Appl. Phys.* **25**, L617 (1986).

73. S. Rauch, J. Sacher, Compact Bragg grating stabilized ridge waveguide laser module with a power of 380 mW at 780 nm. *IEEE Photonics Technol. Lett.* **27**, 1737–1740 (2015).

74. A. Jimenez, T. Milde, N. Staacke, C. Assmann, J. O'Gorman, J. R. Sacher, Narrow-line diode laser packaging and integration in the NIR and MIR spectral range, in *Components and Packaging for Laser Systems III* (SPIE, 2017), vol. 10085, p. 1008505.

75. L. Hildebrandt, R. Knispel, S. Stry, J. R. Sacher, F. Schael, Antireflection-coated blue GaN laser diodes in an external cavity and Doppler-free indium absorption spectroscopy. *Appl. Opt.* **42**, 2110–2118 (2003).

76. W. Chow, S. Koch, *Semiconductor-Laser Fundamentals: Physics of the Gain Materials* (Springer, 1999).

77. A. Bannaron, B. Ranjan, M. Kawasugi, H. Sato, Y. Sakurai, S. Yokoyama, Long-term photoreliability of electro-optic polymer for high optical power modulator, in *2024 IEEE Silicon Photonics Conference (SiPhotonics)* (IEEE, 2024), pp. 1–2.

78. Y. Guo, R. Zhao, G. Zhou, L. Lu, A. Stroganov, M. S. Nisar, J. Chen, L. Zhou, Thermally tuned high-performance III-V/Si_3N_4 external cavity laser. *IEEE Photonics J.* **13**, 1–13 (2021).

79. X. Guo, L. Zhang, L. Chen, J. Liu, T. Liu, S. Zhang, Ultra-narrow linewidth laser system based on intra-cavity electro-optic crystal frequency stabilization of external-cavity diode laser. *Opt. Commun.* **545**, 129635 (2023).

80. Z. Tai, L. Yan, Y. Zhang, X. Zhang, W. Guo, S. Zhang, H. Jiang, Electro-optic modulator with ultra-low residual amplitude modulation for frequency modulation and laser stabilization. *Opt. Lett.* **41**, 5584–5587 (2016).

81. C. Salomon, D. Hils, J. L. Hall, Laser stabilization at the millihertz level. *J. Opt. Soc. Am. B* **5**, 1576–1587 (1988).

82. T. Day, E. K. Gustafson, R. L. Byer, Sub-hertz relative frequency stabilization of two-diode laser-pumped Nd:YAG lasers locked to a Fabry-Perot interferometer. *IEEE J. Quantum Electron.* **28**, 1106–1117 (2002).

83. K. Koshelev, S. Lepeshov, M. Liu, A. Bogdanov, Y. Kivshar, Asymmetric metasurfaces with high-*Q* resonances governed by bound states in the continuum. *Phys. Rev. Lett.* **121**, 193903 (2018).

84. M. Lawrence, D. R. Barton III, J. Dixon, J.-H. Song, J. van de Groep, M. L. Brongersma, J. A. Dionne, High quality factor phase gradient metasurfaces. *Nat. Nanotechnol.* **15**, 956–961 (2020).

85. E. Klopfer, S. Dagli, D. Barton III, M. Lawrence, J. A. Dionne, High-quality-factor silicon-on-lithium niobate metasurfaces for electro-optically reconfigurable wavefront shaping. *Nano Lett.* **22**, 1703–1709 (2022).

86. G. Soma, K. Ariu, S. Karakida, Y. Tsubai, T. Tanemura, Subvolt high-speed free-space modulator with electro-optic metasurface. *Nat. Nanotechnol.* **20**, 1625–1632 (2025).

87. Y. Zhang, C. Fowler, J. Liang, B. Azhar, M. Y. Shalaginov, S. Deckoff-Jones, S. An, J. B. Chou, C. M. Roberts, V. Liberman, M. Kang, C. Rios, K. A. Richardson, C. Rivero-Baleine, T. Gu, H. Zhang, J. Hu, Electrically reconfigurable non-volatile metasurface using low-loss optical phase-change material. *Nat. Nanotechnol.* **16**, 661–666 (2021).

88. X. Sha, K. Du, Y. Zeng, F. Lai, J. Yin, H. Zhang, B. Song, J. Han, S. Xiao, Y. Kivshar, Q. Song, Chirality tuning and reversing with resonant phase-change metasurfaces. *Sci. Adv.* **10**, eadn9017 (2024).

89. A. Barreda, C. Zou, A. Sinelnik, E. Menshikov, I. Sinev, T. Pertsch, I. Staude, Tuning and switching effects of quasi-BIC states combining phase change materials with all-dielectric metasurfaces. *Opt. Mater. Express* **12**, 3132–3142 (2022).

90. D. Salac, W. Lu, C.-W. Wang, A. M. Sastry, Pattern formation in a polymer thin film induced by an in-plane electric field. *Appl. Phys. Lett.* **85**, 1161–1163 (2004).

91. F. Closa, F. Ziebert, E. Raphaël, Interplay of internal stresses, electric stresses, and surface diffusion in polymer films. *Phys. Rev. E* **83**, 051603 (2011).

92. R. Verma, A. Sharma, K. Kargupta, J. Bhaumik, Electric field induced instability and pattern formation in thin liquid films. *Langmuir* **21**, 3710–3721 (2005).

93. E. Schäffer, T. Thurn-Albrecht, T. P. Russell, U. Steiner, Electrically induced structure formation and pattern transfer. *Nature* **403**, 874–877 (2000).

94. L. F. Pease III, W. B. Russel, Electrostatically induced submicron patterning of thin perfect and leaky dielectric films: A generalized linear stability analysis. *J. Chem. Phys.* **118**, 3790–3803 (2003).

95. J. Grgić, E. Campaioli, S. Raza, P. Bassi, N. A. Mortensen, Coupled-resonator optical waveguides: *Q*-factor and disorder influence. *Opt. Quantum Electron.* **42**, 511–519 (2011).

96. B. E. A. Saleh, M. C. Teich, *Fundamentals of Photonics* (Wiley, 2019).

97. K. D. Singer, M. G. Kuzyk, J. E. Sohn, Second-order nonlinear-optical processes in orientationally ordered materials: Relationship between molecular and macroscopic properties. *J. Opt. Soc. Am. B* **4**, 968–976 (1987).

98. N. Yu, F. Capasso, Flat optics with designer metasurfaces. *Nat. Mater.* **13**, 139–150 (2014).
